# A Prognostic Risk Model for Hepatocellular Carcinoma Integrating Ferroptosis and Metabolic Reprogramming Signatures

**DOI:** 10.7150/jca.135837

**Published:** 2026-07-10

**Authors:** Hao Ling, Yanzhu Hu

**Affiliations:** Department of Surgery, Klinikum rechts der Isar, TUM School of Medicine and Health, Technical University of Munich, Munich, Germany.

**Keywords:** hepatocellular carcinoma, ferroptosis, metabolic reprogramming, prognostic model, immune checkpoint genes

## Abstract

**Background:**

Hepatocellular carcinoma (HCC) continues to impose a heavy global health burden, with high incidence and mortality. The disease is highly heterogeneous and is commonly detected at late stages, which compromises treatment outcomes. Ferroptosis and metabolic reprogramming are increasingly recognized as key processes in HCC development; however, their roles in disease progression and therapeutic response remain incompletely understood. This research aimed to identify genes related to ferroptosis and metabolic reprogramming (FPMRRGs) that may serve as putative biomarkers and therapeutic targets in HCC.

**Methods:**

The Cancer Genome Atlas (TCGA), including 369 HCC specimens and 50 normal controls, along with two Gene Expression Omnibus (GEO) datasets (GSE10143 and GSE76427), were analyzed using R (v4.3.3). From a curated list of 451 FPMRRGs, differentially expressed genes (DEGs) between tumor and normal tissues were identified. Univariate Cox regression analysis was then conducted to explore their prognostic relevance and to define molecular subtypes of HCC. Specimens were categorized into 2 subtypes using ConsensusClusterPlus, and overall survival differences were evaluated via survival analysis. Functional and pathway enrichment analyses were conducted to investigate the functional roles of these genes. Immune-related features were evaluated using the Mann-Whitney U test. A prognostic risk model was constructed using least absolute shrinkage and selection operator (LASSO) regression followed by multivariate Cox analysis. Model performance was assessed using receiver operating characteristic (ROC) curves and calibration plots. Immune cell infiltration was estimated by single-sample GSEA, and pathway activity differences were examined using gene set variation analysis (GSVA).

**Results:**

HCC specimens were divided into 2 molecular subtypes, which demonstrated obvious differences in overall survival and immune-related features, including immune checkpoint gene expression and tumor immune dysfunction and exclusion (TIDE) scores. A prognostic model based on 12 key FPMRRGs demonstrated good predictive performance for 1- and 3-year overall survival, with moderate performance for 5-year survival. The prognostic value and expression patterns of these genes were further validated across independent datasets. In addition, these genes were mainly enriched in pathways linked to fatty acid metabolism and HIF-1 signaling, and were closely associated with patterns of immune cell infiltration.

**Conclusions:**

This study identified numerous key genes linked to ferroptosis and metabolic reprogramming in HCC and developed a robust prognostic risk model. Our results offer new insight into the molecular basis of HCC and highlight potential biomarkers for more individualized treatment approaches. Further studies, particularly those combining clinical validation with functional experiments, are required to verify these findings and examine the therapeutic potential of targeting these pathways.

## Introduction

Hepatocellular carcinoma (HCC) is the most common type of primary liver cancer and remains a major global health concern due to its high incidence and mortality. It accounts for nearly 90% of all liver cancer cases worldwide, with increasing rates driven largely by chronic liver diseases such as viral hepatitis, alcohol-related liver disease, and, more recently, metabolic dysfunction-associated steatohepatitis 1. Managing HCC remains difficult, as the disease is often aggressive, diagnosed at an advanced stage, and frequently resistant to conventional therapies 2, 3. Although progress has been made in surgical resection, liver transplantation, and locoregional treatments, outcomes for patients with advanced disease are still poor. This is partly due to the complex and multifactorial nature of HCC, which involves genetic, environmental, and metabolic factors. Together, these challenges highlight the need to identify reliable biomarkers and develop more effective targeted therapies 4, 5.

Recently, increasing attention has been paid to the molecular and metabolic changes that drive HCC development. Ferroptosis, an iron-dependent form of regulated cell death driven by lipid peroxidation, has gained increasing attention in cancer research 6, 7. In HCC, ferroptosis has been linked to tumor progression, shaping of the tumor microenvironment, and responses to therapy, including immunotherapy 8-10. At the same time, metabolic reprogramming allows tumor cells to adapt to changing conditions by reshaping energy production and biosynthetic pathways, thereby supporting tumor growth and survival. This phenomenon is well recognized across cancers and has also been described in liver malignancies 11, 12. Several recent studies have focused specifically on metabolic alterations in HCC, particularly their interaction with the immune system 13-18. In addition, disruptions in iron metabolism and lipid processing have been shown to contribute to tumor progression and treatment resistance 19, 20. Despite these advances, the relationship between ferroptosis and metabolic reprogramming in HCC is still not fully understood, and their combined impact across different HCC subtypes remains unclear.

Herein, we sought to identify key genes related to ferroptosis and metabolic reprogramming that are associated with HCC heterogeneity and progression. Using transcriptomic data from The Cancer Genome Atlas (TCGA) and Gene Expression Omnibus (GEO), we carried out a comprehensive bioinformatics analysis to identify relevant genes. In accordance with these findings, we constructed a prognostic risk model using least absolute shrinkage and selection operator (LASSO) regression combined with Cox analysis to stratify patients according to survival risk. We also examined immune checkpoint gene expression to better characterize the interplay between tumor metabolism, ferroptosis, and immune regulation. A set of 12 key genes was identified, showing strong associations with both patient survival and the immune landscape of HCC. These genes may act as useful biomarkers for patient stratification and offer potential targets for more precise therapeutic strategies aimed at improving clinical outcomes.

## Materials and Methods

### Dataset Acquisition and Preprocessing

We obtained HCC transcriptomic data from TCGA via TCGAbiolinks (v2.30.0) 21 (https://portal.gdc.cancer.gov/). Following exclusion of patients with missing clinical information, 369 HCC tumor specimens and 50 normal control specimens were retained for analysis. Additional datasets, GSE10143 and GSE76427, comprising 195 HCC specimens and 359 control specimens, were downloaded from the GEO using the GEOquery package 22 (https://www.ncbi.nlm.nih.gov/geo/) (Table 1). All datasets were normalized and annotated using the limma package (v3.58.1) 23. Ferroptosis-related genes were collected from multiple sources. First, 2,023 genes were obtained from GeneCards. In addition, a literature-based search on PubMed using the keyword “ferroptosis” 24 yielded 144 genes. After merging these lists and removing duplicates, 2,059 ferroptosis-related genes were identified. Similarly, metabolic reprogramming-related genes (MRRGs) were collected by searching “metabolic reprogramming” and retaining only protein-coding genes, resulting in 1,951 genes. An additional literature-based search 25 identified 9 more MRRGs, and after merging and removing duplicates, 1,954 MRRGs were obtained (Table S1). Comparing the ferroptosis and metabolic reprogramming lists, we identified 451 genes associated with both processes, referred to as ferroptosis- and metabolic reprogramming-related genes (FPMRRGs). Intersecting these with the TCGA-LIHC DEGs yielded 333 ferroptosis- and metabolic reprogramming-related differentially expressed genes (FPMRRDEGs).

### Identification of Differentially Expressed FPMRRDEGs in HCC

The TCGA-LIHC dataset was assigned to HCC and control groups. Differentially expressed genes (DEGs) between tumor and normal specimens were identified using DESeq2 26 with thresholds of |log2 fold-change| > 0.25 and *p* < 0.05. Genes with log2 fold-change > 0.25 were considered upregulated, whereas genes with log2 fold-change < -0.25 were considered downregulated. The results were visualized using volcano plots produced with ggplot2. FPMRRDEGs associated with HCC were determined by overlapping DEGs with the FPMRRGs. Univariate Cox regression analysis (*p* < 0.05) was then applied to identify genes relevant to HCC prognosis, which were used for subsequent subtype classification. A sensitivity analysis varying the |log2 fold-change| cutoff from 0.25 to 1.5 confirmed that the 12-gene panel is preserved at 0.25; at stricter cuts of 0.5, 1.0, and 1.5, 11, 6, and 5 genes are retained respectively, with refit Cox C-indices of 0.712, 0.698, and 0.694 versus 0.710 for the published 12-gene model (Table S6, Table S6b, Figure S6).

### Identification of HCC Subtypes

Consensus clustering 27 was performed on the TCGA-LIHC dataset using expression profiles of FPMRRGs to identify HCC subtypes. Two clusters were defined, with the process repeated 50 times using 80% of the samples to ensure stability. Gene expression differences between subtypes were visualized with heatmaps, and overall survival differences were examined via Kaplan-Meier (KM) analysis.

### Differential Expression Analysis and GSEA of HCC Subtypes

Using the TCGA-LIHC dataset, DESeq2 was applied to compare gene expression levels between HCC subtypes and normal liver tissue. DEGs were defined using thresholds of |log2 fold-change| > 1 and *p* < 0.05. Volcano plots of DEGs were produced with ggplot2 (v3.4.4). Overlapping genes with FPMRRGs were identified via a Venn diagram, yielding the set of cluster_FPMRRDEGs for subsequent analyses. GSEA was performed using clusterProfiler (v4.10.0) to evaluate ranked DEGs based on log2 fold change. Parameters included 2022 seeds, 1,000 permutations, and gene sets containing 10-500 genes, using the c2 collection from MSigDB 28. Significance was defined as adjusted *p* < 0.05 and false discovery rate (FDR) < 0.05. Functional enrichment of FPMRRDEGs was further assessed using GO and KEGG analyses in clusterProfiler, with *p* < 0.05 deemed statistically significant.

### Analysis of Immune Activity in HCC Subtypes

Immune checkpoint genes (ICGs) play key roles in modulating immune function and preserving immune balance. A total of 47 ICGs were obtained from the published literature 29 (Table S2). Expression differences of these genes among HCC subtypes were examined using the Mann-Whitney U test in the TCGA-LIHC dataset. The TIDE framework (http://tide.dfci.harvard.edu) was used to evaluate immune function and exclusion scores in each HCC subtype. Differences in TIDE scores between subtypes were analyzed with Mann-Whitney U 30 (https://www.cbioportal.org/), and differences in TMB and MSI across subtypes were similarly tested with Mann-Whitney U. Immunogenicity scores were calculated using the Immuno-Oncology Biological Research (IOBR) R package (v0.99.9), and subtype-specific differences were examined with Mann-Whitney U.

### Establishment of a Prognostic Model for HCC

A prognostic model was established in accordance with FPMRRG expression. Candidate genes were first screened by univariable analysis and then refined using LASSO for variable selection. Patients were stratified into risk groups on the basis of the resulting RiskScore, and overall survival differences were examined via KM analysis. The predictive performance of the model was assessed with time-dependent ROC curves to compute the AUC values for 1/3/5-year survival. Calibration curves and decision curve analysis (DCA) were further employed to assess model precision and clinical applicability. Internal model stability was further assessed by 10-fold cross-validation × 10 repeats and by 1000-iteration bootstrap optimism correction (Harrell's enhanced bootstrap).

### KM Curve Validation and Expression Analysis of Key Genes

To confirm the robustness of the identified HCC subtypes and their associated overall survival differences, KM survival analyses were conducted with *survival* (v3.5-7) on the TCGA, GSE10143, and GSE76427 datasets. In addition, DEG expression levels were evaluated between HCC patients and healthy subjects in the GSE10143 and GSE76427 datasets using group comparison plots to visualize differential expression.

### Immune activity Analysis in HCC and Normal Samples

ssGSEA was implemented on the TCGA dataset to quantify the abundance of immune cell populations, generating an immune infiltration matrix for HCC. Expression differences between HCC and healthy subjects were visualized with ggplot2, allowing identification of immune cell populations with significant variation. Spearman correlation analysis was applied to measure relationships among immune cells, as well as between key genes and immune cell populations. Correlation results were visualized using ggplot2 (v3.4.4) and pheatmap (v1.0.12).

### Functional immune signature scoring and single-cell validation

To evaluate functional immune states beyond cell-type abundance, we applied the singscore package to compute rank-based enrichment scores for 11 curated functional immune gene sets (including effector function, antigen presentation, and suppressive states) in TCGA-LIHC samples. Gene sets were derived from published literature and the MSigDB database. Differences in signature scores between low- and high-risk groups (defined by median RiskScore) were assessed using the Wilcoxon rank-sum test with Benjamini-Hochberg correction for multiple testing.

For single-cell validation, we utilized the GSE140228 dataset (15,319 CD45+ tumour-infiltrating immune cells from four HCC patients) retrieved via the TISCH2 database. Cell-type annotation was obtained directly from TISCH2. Expression of the 12 signature genes was visualized across major immune lineages and 28 finer subtypes using dot plots. No additional data processing or re-annotation was performed beyond the published TISCH2 annotations.

### GSVA of Low- and High-risk HCC Subtypes

GSVA, a nonparametric approach, was applied to evaluate pathway enrichment in TCGA and GEO gene expression matrices. Using the H.all.v7.4.Symbols.gmt gene set from MSigDB (v1.50.0), HCC specimens were stratified into low- and high-risk groups. GSVA was used to identify pathways and genes enriched in the high-risk group relative to the low-risk group, with significance defined as *p* < 0.05.

### Statistical Analysis

All statistical tests were implemented through R (v4.3.3). Continuous data are expressed as mean±standard deviation (SD). Two-group comparisons were conducted with Wilcoxon test, whereas comparisons among three or more groups used the Kruskal-Wallis test. Categorical data were evaluated using Fisher's exact or chi-square tests, as appropriate. Correlations between variables were calculated using Spearman's method unless otherwise specified. *P*<0.05 was deemed statistically significant. The proportional hazards assumption of the multivariate Cox model was assessed using Schoenfeld residuals. Although several individual covariates showed violation of the proportional hazards assumption, the global test was non-significant (p = 0.171), supporting the overall validity of the model (Table S7b).

## Results

### Technology Roadmap

**Figure 1:**
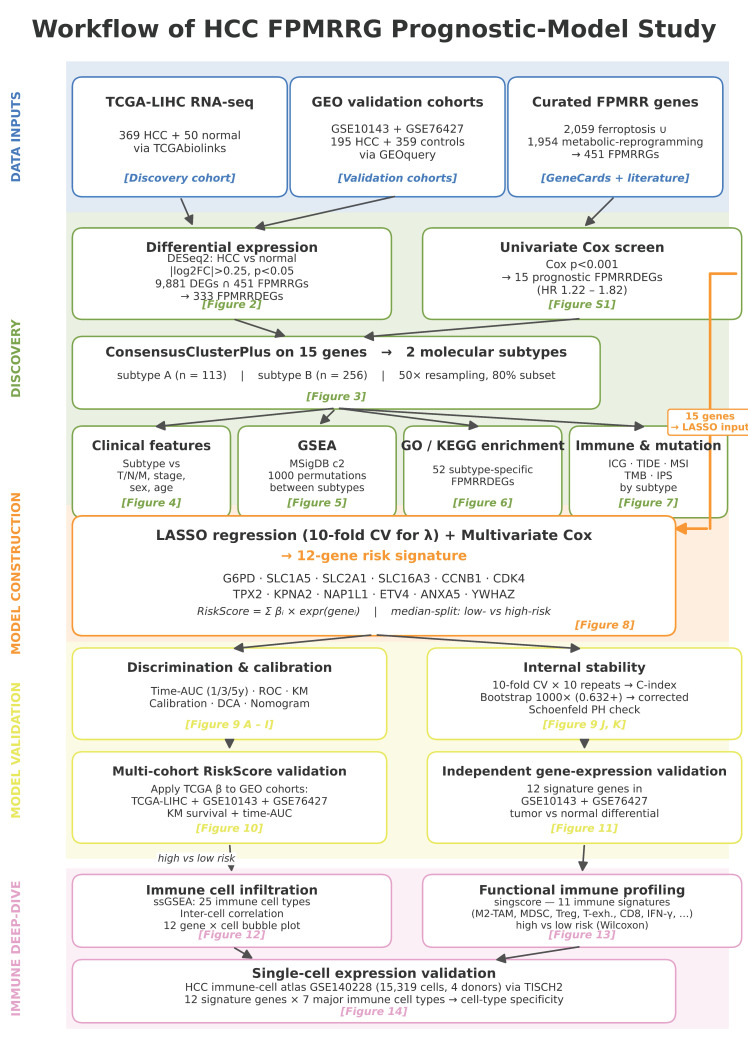
Technology roadmap.

The overall study design and analysis workflow are summarized in Figure 1.

### FPMRRGs Are Differentially Expressed in HCC

**Figure 2:**
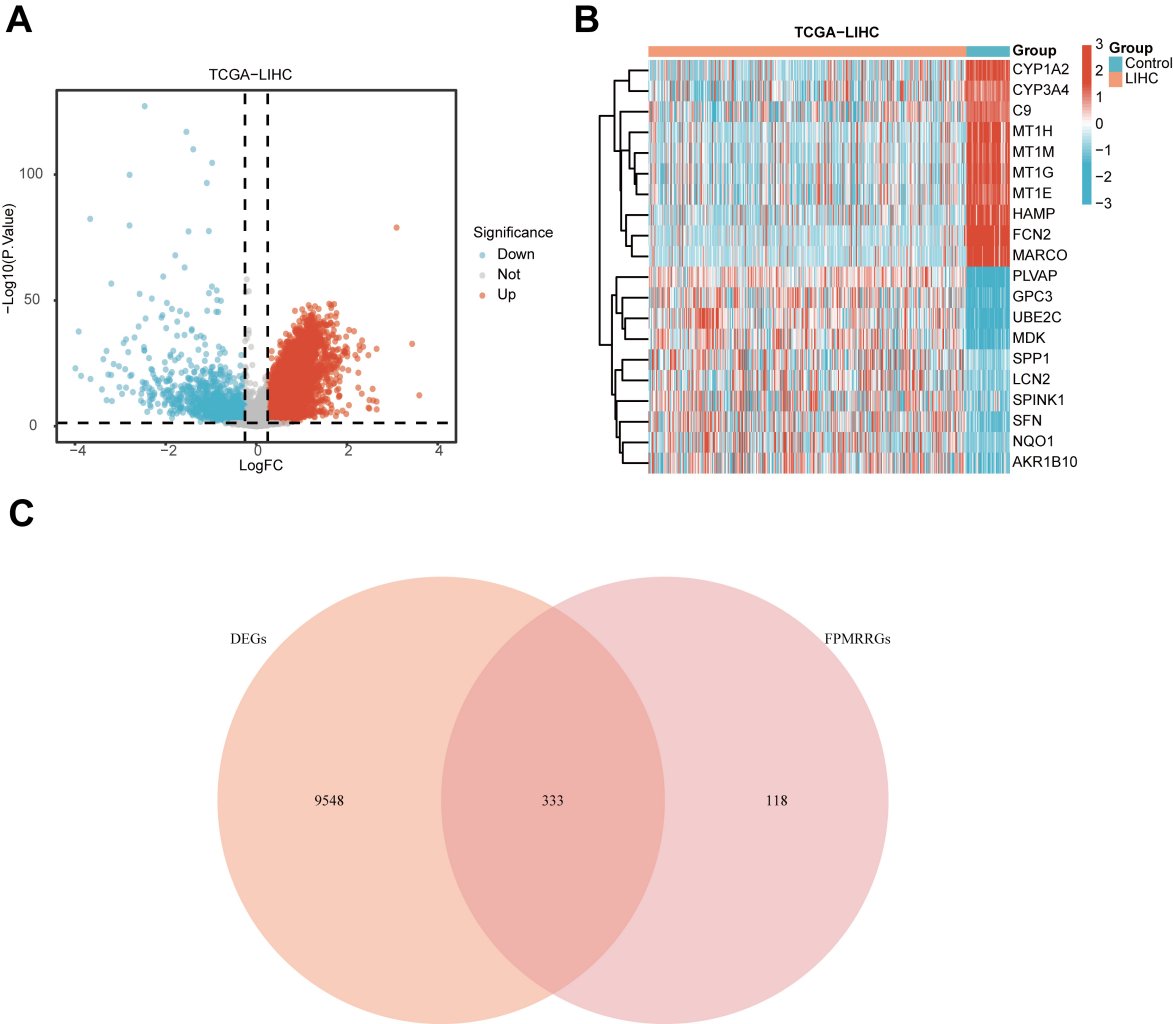
Differential expression of FPMRRGs in HCC. (A) Volcano plot showing gene expression differences between HCC and control samples in the TCGA-LIHC dataset. (B) Heatmap of the top 20 DEGs. (C) Venn diagram revealing the overlap between TCGA-LIHC DEGs and FPMRRGs. TCGA: The Cancer Genome Atlas; LIHC: liver cancer; FPMRRGs: ferroptosis- and metabolic reprogramming-related genes.

To identify HCC-associated FPMRRGs, we first examined differences in gene expression between tumor and healthy liver tissues. Overall, 9,881 DEGs were identified, involving 1,219 downregulated and 8,662 upregulated genes (Figure 2A). The top 20 DEGs are shown in the heatmap (Figure 2B). We then compiled a list of 451 genes related to both ferroptosis and metabolic reprogramming based on data from GeneCards and the published literature. Intersecting this list with the TCGA-LIHC DEGs yielded 333 overlapping genes (FPMRRDEGs) (Figure 2C). Among these, 15 genes with *p* < 0.001 (Table S3) were further analyzed using univariate Cox regression (Figure S1). The hazard ratios for these genes ranged from 1.22 to 1.82. Overall, these results indicate that a subset of genes involved in ferroptosis and metabolic reprogramming is significantly dysregulated in HCC compared with normal liver tissue.

### Distinct HCC Subtypes Defined by Ferroptosis- and Metabolic Reprogramming-related Genes Show Different Clinical Features and Outcomes

**Figure 3:**
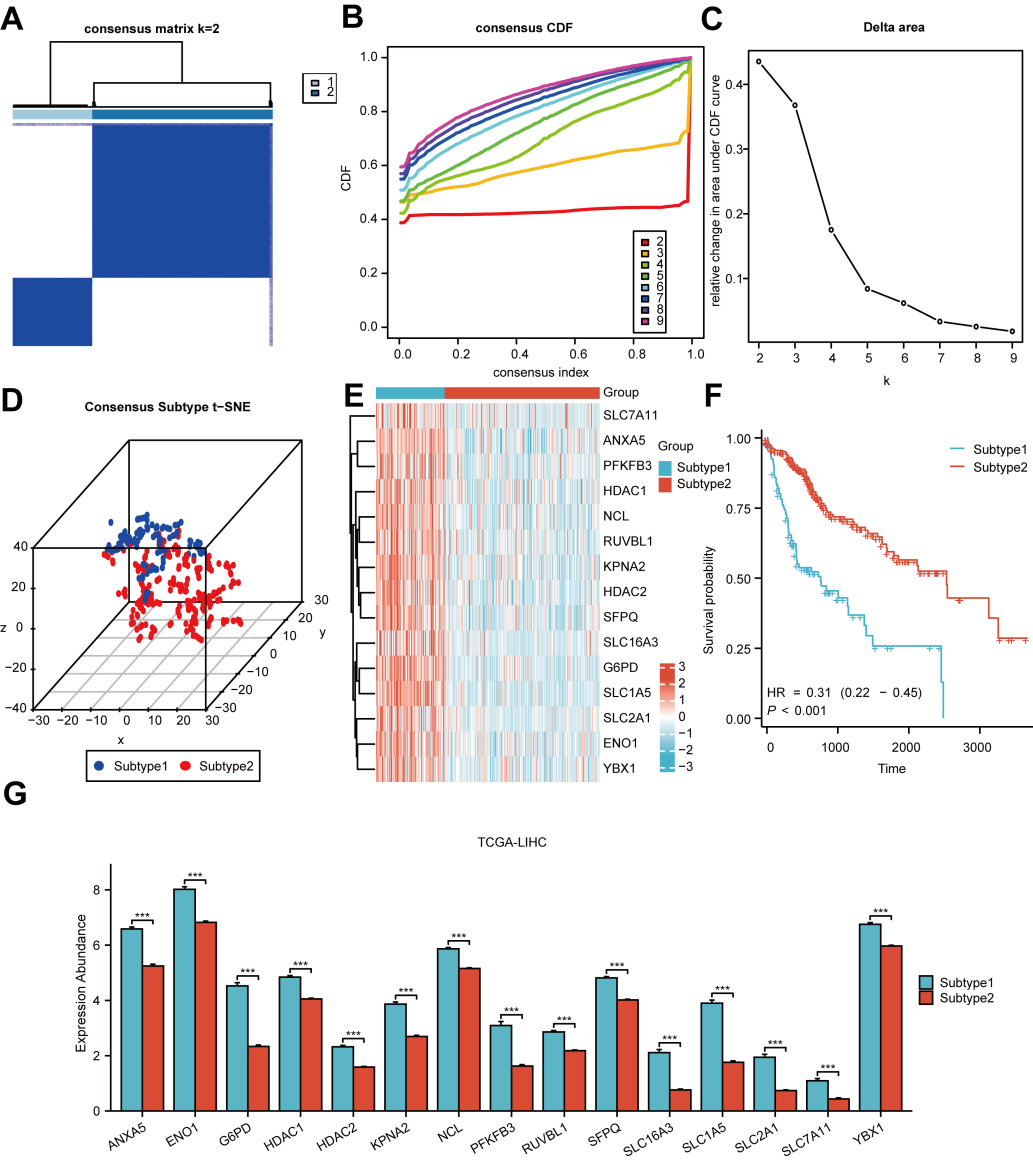
Consensus clustering identifies two molecular subtypes of HCC. (A) Consensus clustering of HCC samples. (B) Cumulative distribution function (CDF) plot of clustering results. (C) Delta area plot displaying the relative changes in CDF. (D) Three-dimensional t-SNE plot illustrating the distribution of HCC subtypes. (E) Heatmap revealing the expression of 15 FPMRRDEGs across the two subtypes. (F) KM analysis of overall survival for patients in the two subtypes. (G) Expression levels of the 15 FPMRRDEGs in each subtype. t-SNE: t-distributed stochastic neighbor embedding; CDF: cumulative distribution function; HR: hazard ratio; HCC: hepatocellular carcinoma. ***p < 0.001.

**Figure 4:**
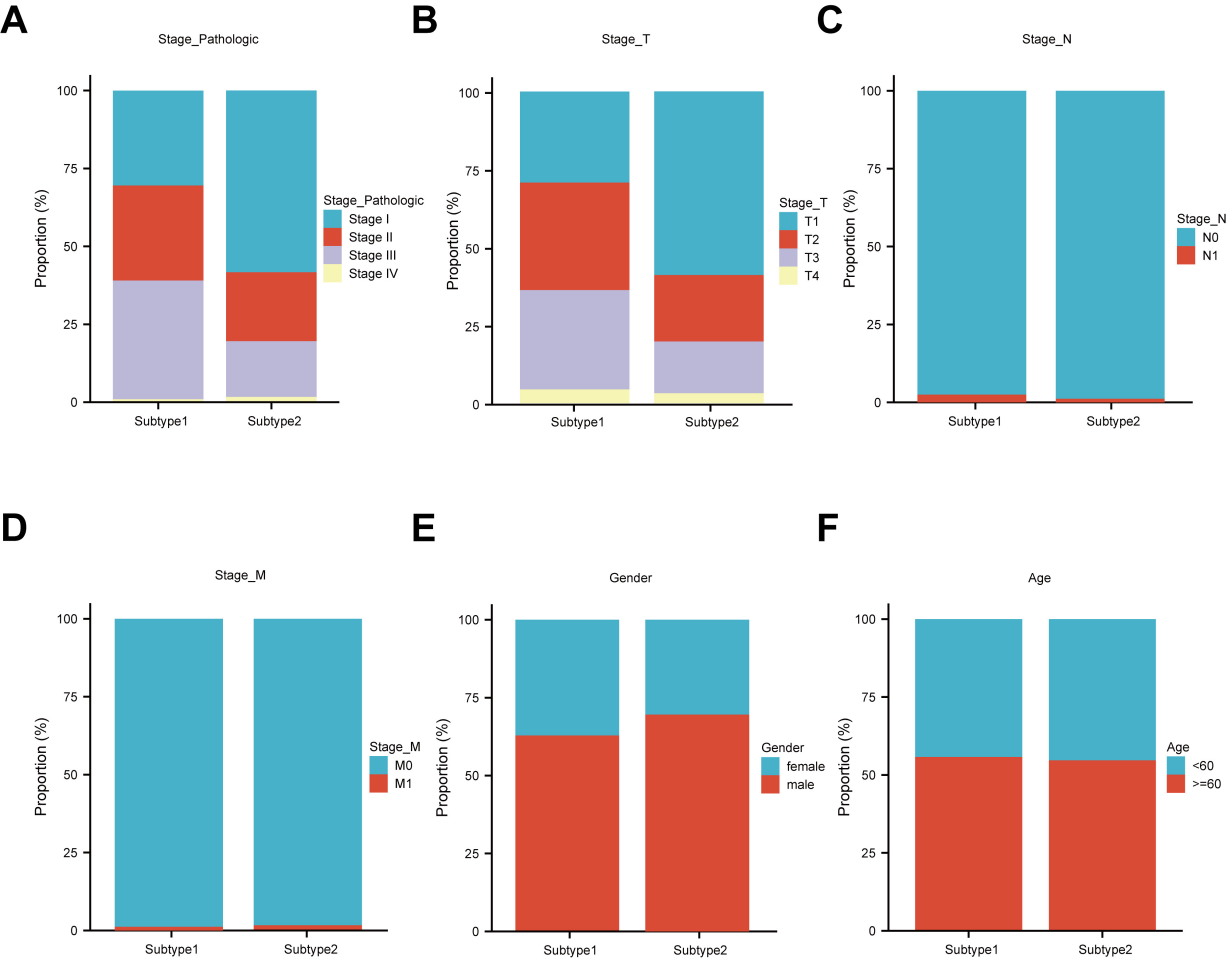
Clinical characteristics differ between the two HCC subtypes. Distribution of patients with subtype 1 and subtype 2 according to pathologic stage (A), T stage (B), N stage (C), M stage (D), gender (E), and age (F).

We next examined whether expression patterns of FPMRRGs varied across HCC samples. Using the 15 prognostically significant FPMRRDEGs (*p* < 0.001), consensus clustering was implemented on the TCGA-LIHC cohort. This analysis identified two distinct subtypes: subtype 1 (*n* = 113) and subtype 2 (*n* = 256) (Figure 3A-C). The clustering results are further illustrated by a three-dimensional t-SNE plot (Figure 3D). A heatmap highlighting differences in gene expression between the 2 subtypes is shown in Figure 3E. Subsequent analysis confirmed that the 15 genes used for clustering were differentially expressed between the two subtypes (Figure 3G). To assess the clinical relevance of these subtypes, KM survival analysis was performed, revealing a significant difference in overall survival between the two groups (Figure 3F). We also compared clinical features between the subtypes (Table 2). Significant differences were observed in pathologic stage and T stage distribution, whereas no obvious differences were noted for N stage, M stage, gender or age (Figure 4). Collectively, these findings imply that HCC can be stratified into biologically distinct subtypes based on ferroptosis- and metabolic reprogramming-related gene expression, and that these subtypes differ in both clinical features and patient outcomes.

### Differentially Expressed FPMRRGs between HCC Subtypes are Enriched in Key Biological Functions and Pathways

**Figure 5:**
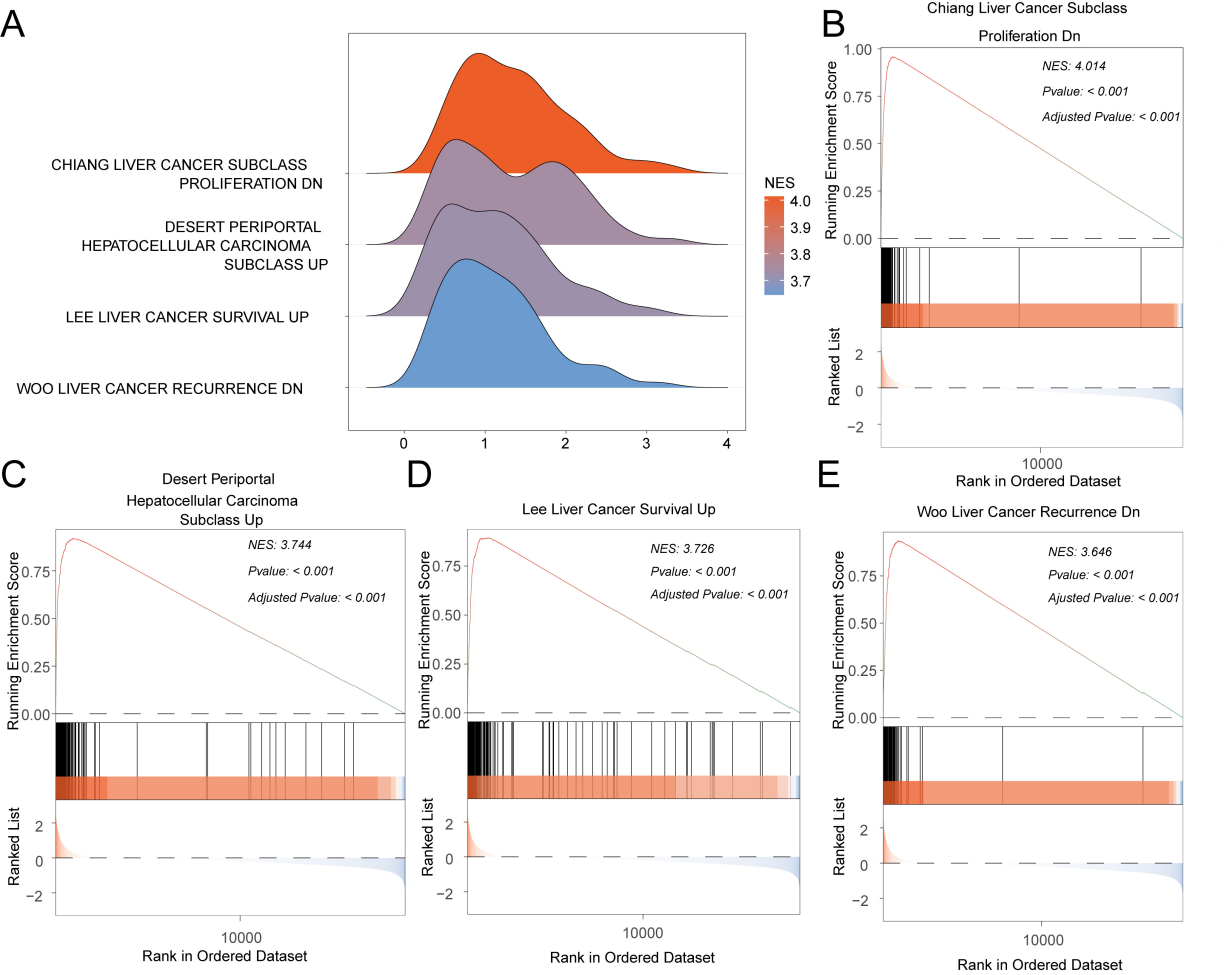
Enrichment of key biological pathways in HCC subtypes. (A) GSEA enrichment plot highlighting significantly enriched biological processes. (B-E) Enrichment plots for representative gene sets: CHIANG_LIVER_CANCER_SUBCLASS_PROLIFERATION_DN (B), DESERT_PERIPORTAL_HEPATOCELLULAR_CARCINOMA_SUBCLASS_UP (C), LEE_LIVER_CANCER_SURVIVAL_UP (D), and WOO_LIVER_CANCER_RECURRENCE_DN (E). Significance criteria: adjusted *p*-value < 0.05 and FDR < 0.05 (Benjamini-Hochberg correction). NES: Normalized Enrichment Score; BH: Benjamini-Hochberg.

Given the clinical differences observed between the two HCC subtypes, we next explored the underlying functional differences that might explain these findings. Differential expression analysis between the 2 subtypes in TCGA-LIHC identified a set of DEGs (Table S4), which were subsequently analyzed using GSEA. GSEA indicated that these genes were markedly enriched in pathways and gene sets associated with liver cancer proliferation, periportal HCC, patient survival, and tumor recurrence (Figure 5, Table 3). This suggests that the molecular divergences between the subtypes are closely linked to key features of tumor progression and clinical outcome.

**Figure 6:**
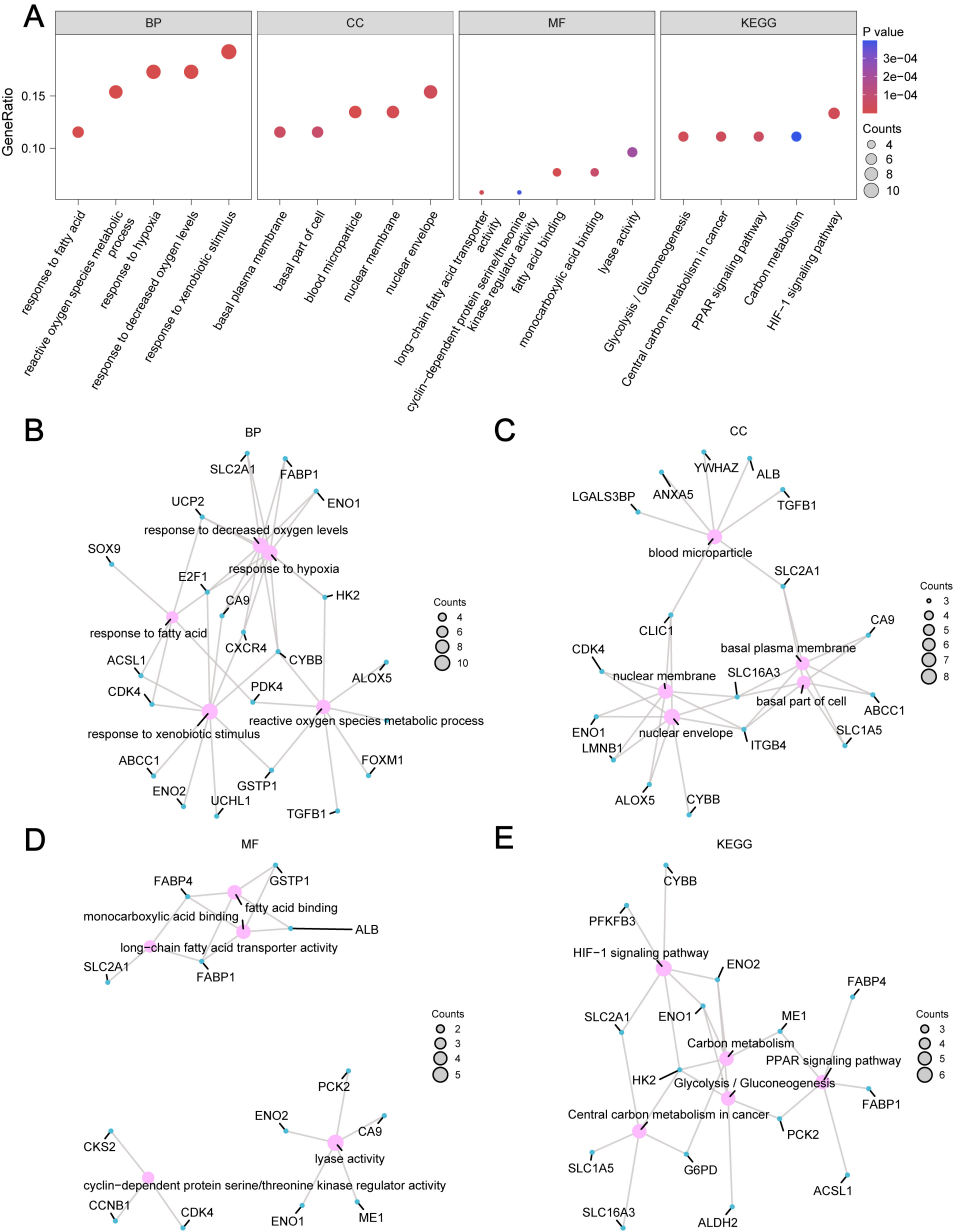
GO and KEGG enrichment analyses of subtype-specific FPMRRDEGs. (A) Bubble plot showing enrichment data for the 52 differentially expressed FPMRRDEGs. (B-E) Network plots of enriched biological processes (B), cellular components (C), molecular functions (D), and KEGG (E) pathways. Node size represents the number of genes, and edges indicate functional associations. Significance threshold: *p* < 0.05.

To further focus on ferroptosis- and metabolic reprogramming-related genes, we intersected the subtype-specific DEGs with the previously defined list of 451 FPMRRGs, yielding 52 overlapping genes (Figure S2). Functional annotation of these genes showed enrichment across pathways related to metabolism, transport, and cellular signaling (Table 4, Figure 6A). Network analysis provided additional insight into these functional categories. Biological process terms were mainly associated with stress responses, while cellular component terms were enriched in nuclear and basolateral cell regions. Molecular function terms were largely related to fatty acid metabolism and enzyme activity, and several key metabolic and signaling pathways were identified in the KEGG analysis (Figure 6B-E). Together, these findings indicate that differences in the expression of FPMRRGs between HCC subtypes are functionally relevant and may contribute to the distinct biological behavior and clinical outcomes observed in these tumors.

### The Two HCC Subtypes Show Distinct Immune Profiles and Mutation Characteristics

**Figure 7:**
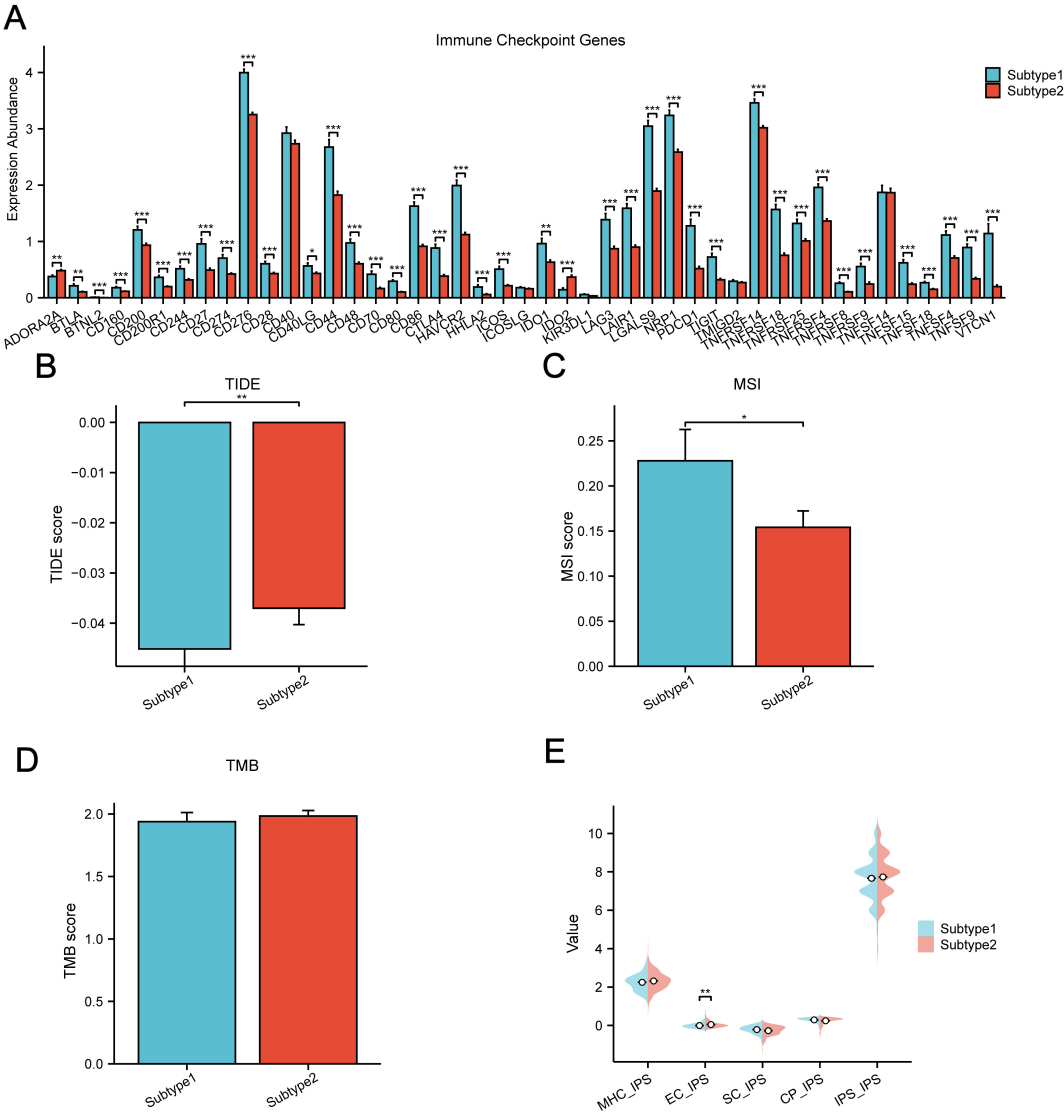
Immune activity and mutation characteristics differ between HCC subtypes. (A) Comparison of ICG expression among subtypes in TCGA-LIHC. (B-D) Comparison of TIDE (B), MSI (C), and TMB (D) scores across the two subtypes. (E) Immunophenoscore (IPS) analysis showing differences in immune-related components between subtypes. ICG: immune checkpoint gene; TIDE: tumor immune dysfunction and exclusion; MSI: microsatellite instability; TMB: tumor mutation burden; IPS: immuno pheno score; EC: effector cells; MHC: major histocompatibility complex; SC: stimulatory cells; CP: checkpoint molecules. *p < 0.05; **p < 0.01; *** *p*< 0.001.

Given the key role of immune regulation and genomic alterations in HCC progression, we next compared immune activity and mutation-related features between the two subtypes. Analysis of ICG expression revealed significant differences between the subtypes, including *ADORA2A*, *BTLA*, and *IDO1* (all *p* < 0.01), as well as *CD40LG* (*p* < 0.05) (Figure 7A). To assess potential responses to immunotherapy, we applied the TIDE algorithm and found an obvious difference in TIDE scores among the groups (*p* < 0.01; Figure 7B), suggesting distinct immune evasion profiles. Immunophenoscore (IPS) analysis further showed significant differences in effector cell activity between the subtypes (*p* < 0.01; Figure 7E). We also examined mutation-related features. Both tumor mutational burden (TMB) and microsatellite instability (MSI) differed remarkably across the subtypes (MSI, *p* < 0.05; Figure 7C; TMB, Figure 7D). Overall, these results indicate that the two HCC subtypes are characterized by distinct immune landscapes and mutation patterns, which may contribute to their different clinical behaviors and treatment responses.

### Development and Verification of a Prognostic Risk Model Based on FPMRRGs

**Figure 8:**
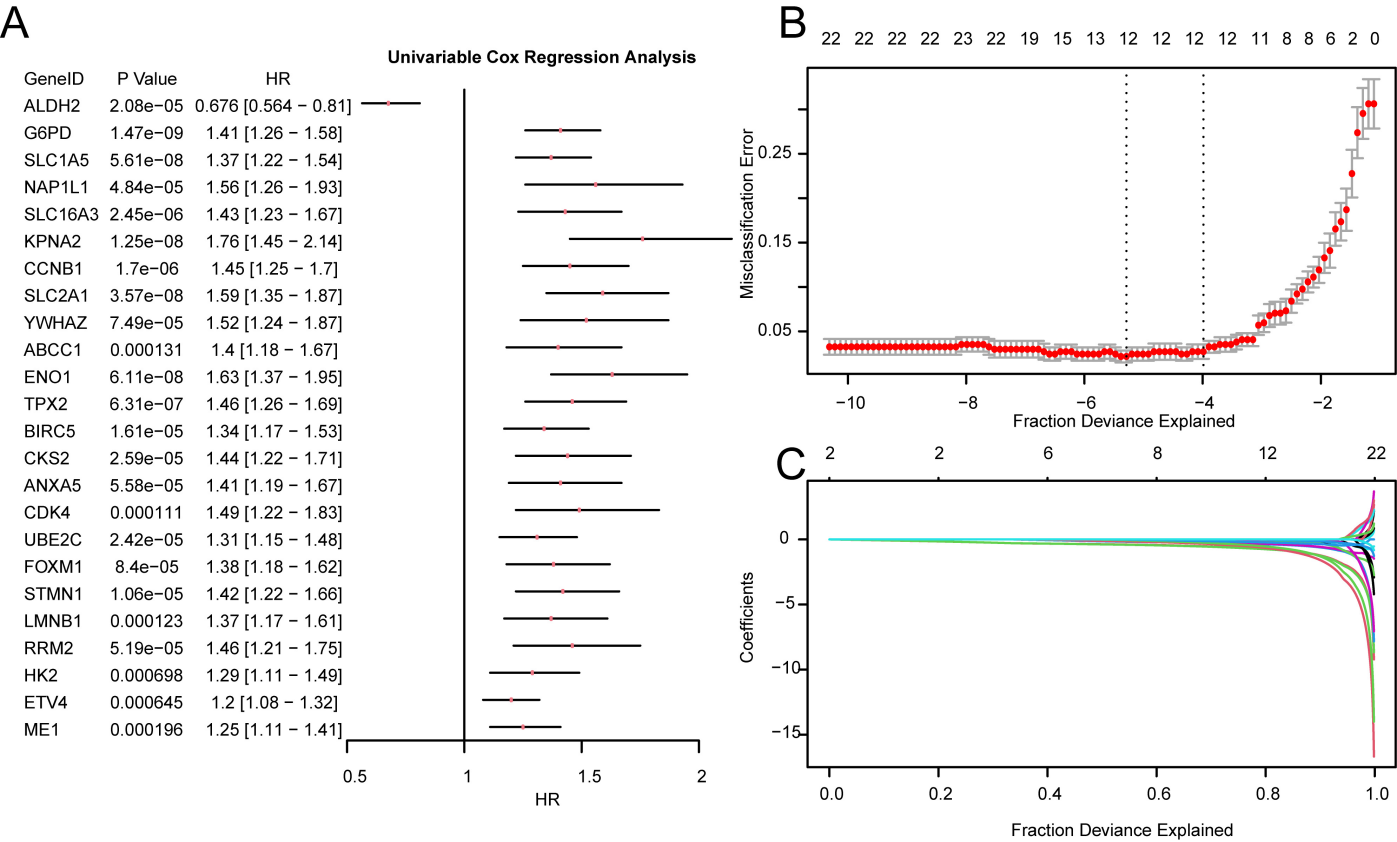
A prognostic model based on FPMRRGs accurately predicts survival in HCC patients. (A) Forest plot of genes identified by univariate Cox regression analysis. (B) Construction of the prognostic risk model using LASSO regression. (C) LASSO coefficient profiles showing variable trajectories. LASSO, least absolute shrinkage and selection operator; HR: hazard ratio.

**Figure 9:**
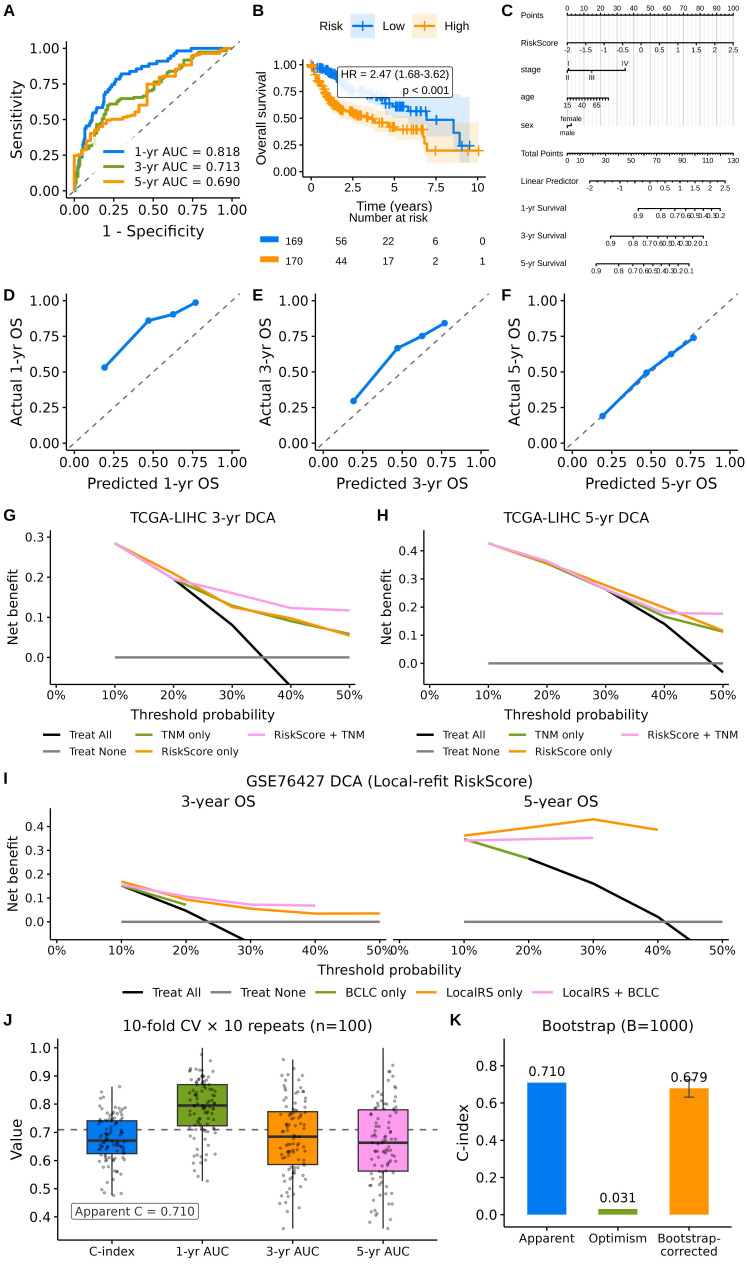
Prognostic model validation. (A) Time-dependent ROC curves for predicting overall survival in HCC. (B) Kaplan-Meier survival analysis comparing low- and high-risk groups. (C) Nomogram integrating risk score and clinical variables based on Cox regression analysis. (D-F) Calibration curves for 1-, 3-, and 5-year survival, respectively. (G-H) Decision curve analysis (DCA) for 3- and 5-year overall survival in TCGA-LIHC, comparing RiskScore alone, AJCC TNM alone, and combined RiskScore + TNM strategies against treat-all and treat-none defaults. (I) DCA for 3- and 5-year overall survival in GSE76427, comparing Local-refit RiskScore alone, BCLC stage alone, and combined Local-refit RiskScore + BCLC strategies. (J) Boxplot of 10-fold cross-validation C-index and 1/3/5-year time-dependent AUC across 100 fold-level estimates (10 folds × 10 repeats) in TCGA-LIHC; the dashed line marks the apparent C-index of 0.710. (K) Apparent C-index versus bootstrap optimism-corrected C-index (B = 1000), showing minimal optimism (0.031) and confirming low overfitting.

Building on the observed roles of ferroptosis and metabolic reprogramming in HCC, we established a prognostic model in accordance with gene expression profiles. Univariate Cox regression (Figure 8A) followed by LASSO regression (Figure 8B, C) identified a 12-gene signature: *G6PD*, *SLC1A5*, *NAP1L1*, *SLC16A3*, *KPNA2*, *CCNB1*, *SLC2A1*, *YWHAZ*, *TPX2*, *ANXA5*, *CDK4*, and *ETV4*. Time-dependent ROC analysis indicated strong predictive performance of the model for 1/3/5-year overall survival (Figure 9A). Calibration curves supported good model calibration, with the highest accuracy at 5 years (Figure 9D-F). DCA further supported the clinical applicability of the model, again showing the strongest performance at 5 years (Figure 9G-I). Internal validation by 10-fold cross-validation × 10 repeats yielded a mean C-index of 0.676 (95% CI 0.491-0.823), and 1000-iteration bootstrap optimism correction yielded a corrected C-index of 0.679 (apparent C = 0.710, optimism = 0.031) (Figure 9J-K, Table S7). The proportional hazards assumption of the multivariate Cox model was assessed using Schoenfeld residuals. Although several individual covariates violated the proportional hazards assumption, the global test was non-significant (p = 0.171), supporting the overall validity of the model (Table S7b). A sensitivity analysis confirmed model robustness across DEG fold-change thresholds (Table S6, Table S6b, Figure S6).

Patients were then stratified into low- and high-risk groups in accordance with the median RiskScore. KM analysis indicated an obvious difference in overall survival between these groups (Figure 9B). GSVA identified multiple pathways that differed between the low- and high-risk groups (Table 5, Figure S3A), and these differences were confirmed through Mann-Whitney U. Notably, pathways such as HALLMARK_BILE_ACID_METABOLISM were significantly enriched (Figure S3B). Quantitative Decision Curve Analysis at the 3-year horizon in TCGA-LIHC showed that the combined RiskScore + TNM model adds a net benefit of approximately +0.06 over TNM alone at threshold 0.50; in GSE76427 at the 5-year horizon, the Local-refit RiskScore adds a net benefit of approximately +0.13 over BCLC alone at threshold 0.20 (Figure 9G-I, Table S9).

A nomogram comprising the LASSO-derived risk scores and clinical variables was established to improve prognostic prediction. The risk score showed stronger predictive value than other clinical factors, whereas age contributed the least (Figure 9C). When compared against AJCC TNM staging in TCGA-LIHC (complete-stage sub-cohort n = 339, 116 events; 24 patients with missing AJCC pathologic stage were excluded to permit paired model comparison), the combined RiskScore + TNM model achieved a C-index of 0.717 (95% CI 0.668-0.765) versus 0.660 (95% CI 0.586-0.734) for TNM alone (paired p = 0.039), with 5-year continuous net reclassification improvement (NRI) = 0.247 (95% CI 0.035-0.442) and integrated discrimination improvement(IDI) = 0.099 (95% CI 0.034-0.170). In GSE76427 (n = 115, events = 23) against BCLC, the Local-refit RiskScore + BCLC model achieved a C-index of 0.812 (95% CI 0.740-0.883) versus 0.719 (95% CI 0.556-0.881) for BCLC alone, with 5-year continuous NRI = 0.621 (95% CI 0.004-1.003) and IDI = 0.374 (95% CI 0.050-0.632) (Table 6).

**Figure 10:**
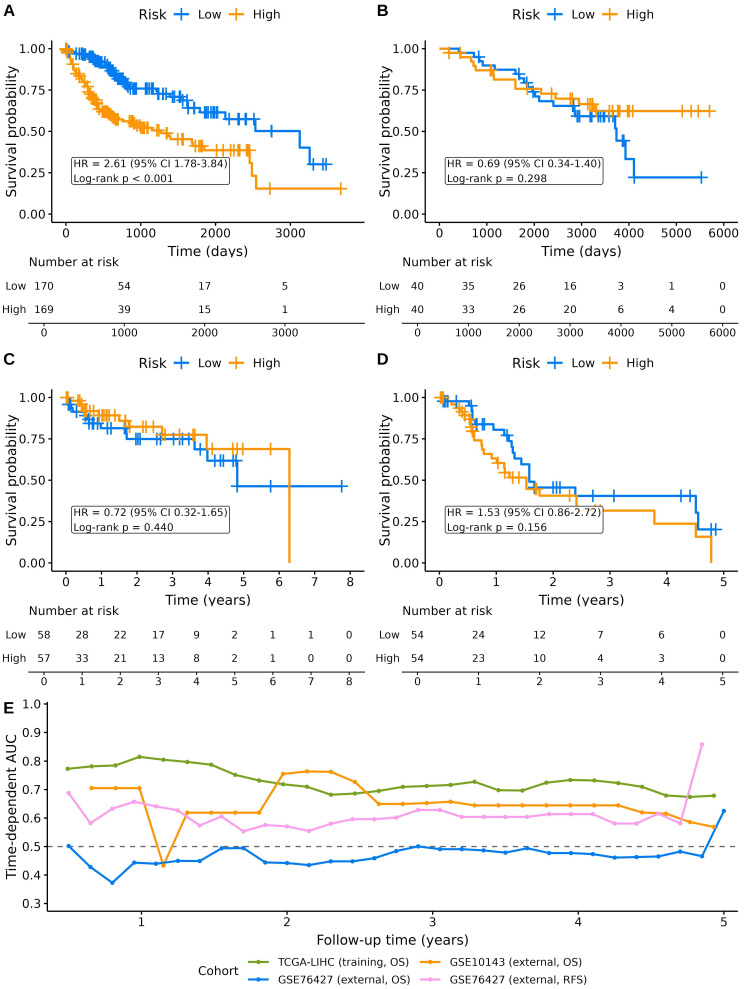
Multi-cohort validation of the 12-gene risk model. (A) Kaplan-Meier curve for the TCGA-LIHC training cohort (n = 363, events = 130) comparing low- and high-risk groups stratified by the median RiskScore. (B-D) External validation in GEO cohorts using cohort-specific local-refit Cox models: (B) GSE10143 overall survival (n = 80, events = 32), (C) GSE76427 overall survival (n = 115, events = 23), and (D) GSE76427 recurrence-free survival (n = 108, events = 48). Hazard ratios with 95% confidence intervals and log-rank p-values are shown in each panel. (E) Time-dependent AUC curves across the three cohorts over 5-year follow-up, demonstrating consistent discriminative performance after cohort-specific recalibration.

To validate the multi-gene risk model in external cohorts, we computed RiskScore in GSE10143 and GSE76427 using two complementary strategies. Direct transfer of TCGA-derived Cox coefficients (TCGA-β) failed to separate risk groups in either cohort (GSE10143 OS HR = 0.69, 95% CI 0.34-1.40, p = 0.30; GSE76427 OS HR = 0.72, 95% CI 0.32-1.65, p = 0.43), indicating that the TCGA-fit weights are not portable across microarray platforms. Local refitting of the 12-gene Cox model within each cohort, however, recovered strong prognostic separation (GSE10143 OS HR = 3.09, 95% CI 1.47-6.51, p = 0.002, C-index = 0.65; GSE76427 OS HR = 13.12, 95% CI 3.70-46.53, p < 0.001, C-index = 0.81; GSE76427 RFS HR = 3.05, 95% CI 1.65-5.67, p < 0.001, C-index = 0.73), confirming that the 12-gene panel carries genuine prognostic signal but requires cohort-specific recalibration (Figure 10, Table S8). Single-gene Kaplan-Meier curves for each of the 12 individual genes in GSE10143 and GSE76427 are provided in Supplementary Figures S4 and S5, respectively.

Taken together, these findings suggest that this 12-gene signature provides a robust prognostic model for HCC and may be useful for risk stratification and guiding clinical decision-making.

### Prognostic Model Genes are Differentially Expressed in HCC and Related to Immune Cell Infiltration

**Figure 11:**
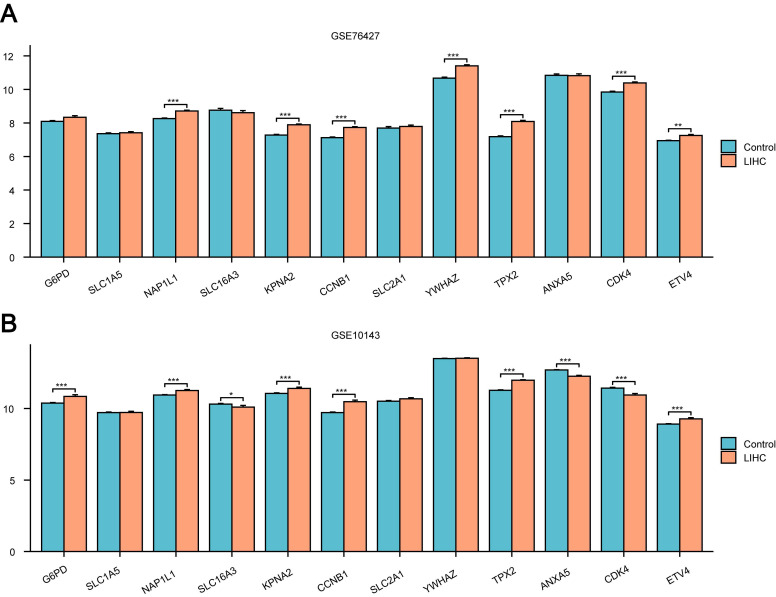
Differential expression of prognostic model genes in HCC and healthy specimens. (A) Gene expression differences between HCC and healthy specimens in GSE76427. (B) Gene expression differences between HCC and healthy specimens in GSE10143. *p < 0.05, **p < 0.01, ***p < 0.001.

We next examined whether the genes included in the prognostic model could also distinguish tumor tissue from normal liver tissue. Expression levels of the 12 key genes (*G6PD*, *SLC1A5*, *NAP1L1*, *SLC16A3*, *KPNA2*, *CCNB1*, *SLC2A1*, *YWHAZ*, *TPX2*, *ANXA5*, *CDK4*, and *ETV4*) were compared between HCC and control samples in the GSE10143 and GSE76427 datasets. In the GSE76427 dataset, significant differences were observed for *NAP1L1*, *KPNA2*, *CCNB1*, *YWHAZ*, *TPX2*, *CDK4*, and *ETV4* (Figure 11A). Similarly, in the GSE10143 dataset, *G6PD*, *NAP1L1*, *KPNA2*, *CCNB1*, *TPX2*, *ANXA5*, *CDK4*, *ETV4*, and *SLC16A3* showed significant differential expression between tumor and normal tissues (Figure 11B). These findings indicate that a substantial proportion of the model genes are consistently dysregulated in HCC.

**Figure 12:**
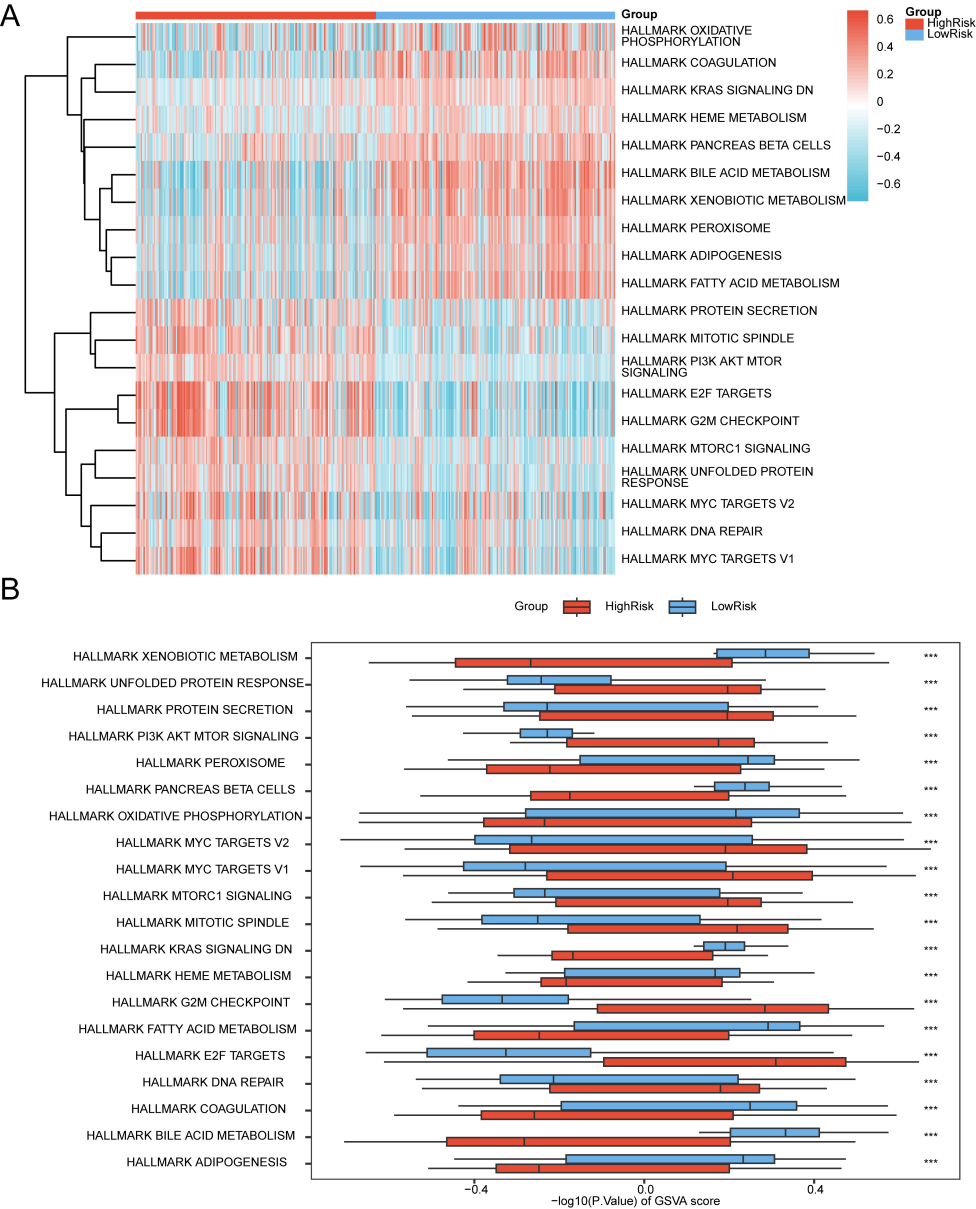
Immune cell infiltration analysis. (A) Comparison of immune cell populations between HCC and control samples using ssGSEA. (B) Correlation heatmap of immune cell infiltration in TCGA-LIHC. (C) Bubble plot showing correlations between key gene expression and immune cell infiltration. Significance levels: ns (*p* ≥ 0.05), *p < 0.05, **p < 0.01, ***p < 0.001.

To explore whether these expression changes were associated with immune activity, immune cell infiltration in TCGA-LIHC was analyzed using ssGSEA. Significant differences were observed in the abundance of 25 immune cell types between HCC and healthy controls (Figure 12A). Correlation analysis revealed complex relationships among these immune cell populations (Figure 12B). Further analysis using a bubble plot demonstrated remarkable associations between key gene expression and immune cell infiltration (Figure 12C). Notably, SLC1A5 expression was positively correlated with activated CD4⁺ T cells (*r* = 0.564), while CCNB1 expression had a negative correlation with eosinophils (*r* = -0.567). This suggests that the genes incorporated in the prognostic model are not only differentially expressed in HCC but are also closely linked to the tumor immune microenvironment.

### Functional Immune Profiling and Single-Cell Validation of the 12-Gene Signature

**Figure 13:**
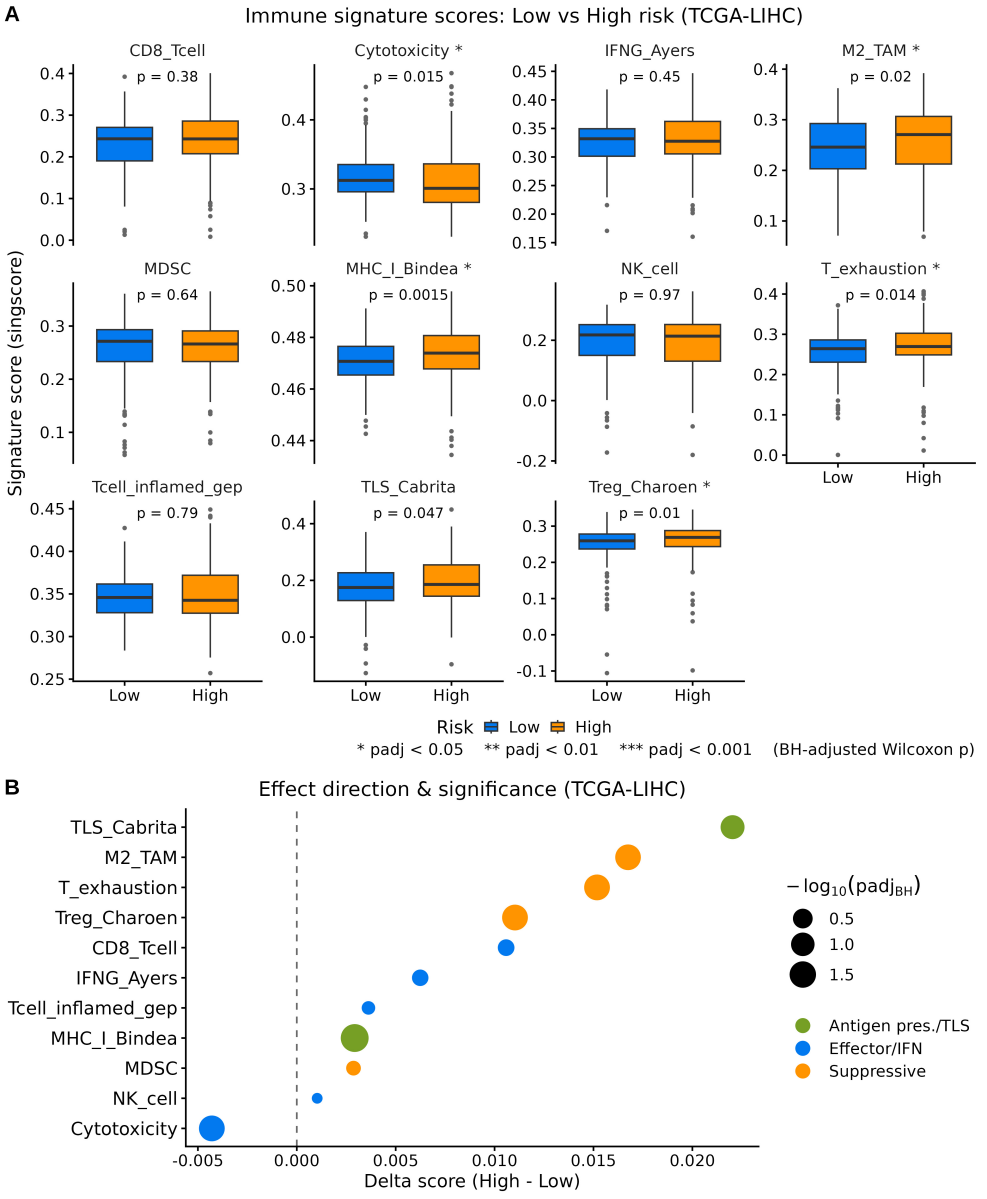
Functional immune profiling of the 12-gene risk model in TCGA-LIHC. (A) Single-sample gene set scoring (singscore) of 11 functional immune signatures (CD8 T cell, cytotoxicity, IFN-γ response, M2 TAM, MDSC, MHC-I, NK cell, T-cell exhaustion, T-cell inflamed GEP, tertiary lymphoid structure, Treg) compared between low- and high-risk groups; boxplots show median and IQR, Wilcoxon p-values are annotated and BH-adjusted significance is indicated (*p < 0.05, **p < 0.01, ***p < 0.001). (B) Bubble plot summarising effect direction and significance of risk-group differences; the x-axis shows the delta score (high minus low), dot size encodes -log10 (adjusted p), and colour groups signatures by functional category (effector/IFN, antigen presentation/TLS, suppressive).

To extend the immune analysis beyond cell-type abundance, we scored 11 functional immune signatures in TCGA-LIHC by singscore^31^ and stratified samples by the median RiskScore. High-risk tumors exhibited significantly elevated MHC class I, Treg, T-cell exhaustion, and M2 TAM signatures, together with significantly reduced cytotoxicity after Benjamini-Hochberg correction (Figure 13A and Table S10). In contrast, MDSC, CD8 T cell, and IFN-γ response signatures showed the same directional changes but did not reach statistical significance after multiple-testing correction. These results indicate that high-risk tumors are characterized by a more exhausted and suppressive immune phenotype, despite relatively preserved MHC class I expression (Wilcoxon BH-adjusted p < 0.05; Figure 13A, Table S10). The bubble plot in Figure 13B summarises the magnitude and direction of these differences, with suppressive signatures positively associated with the risk score and effector signatures negatively associated.

**Figure 14:**
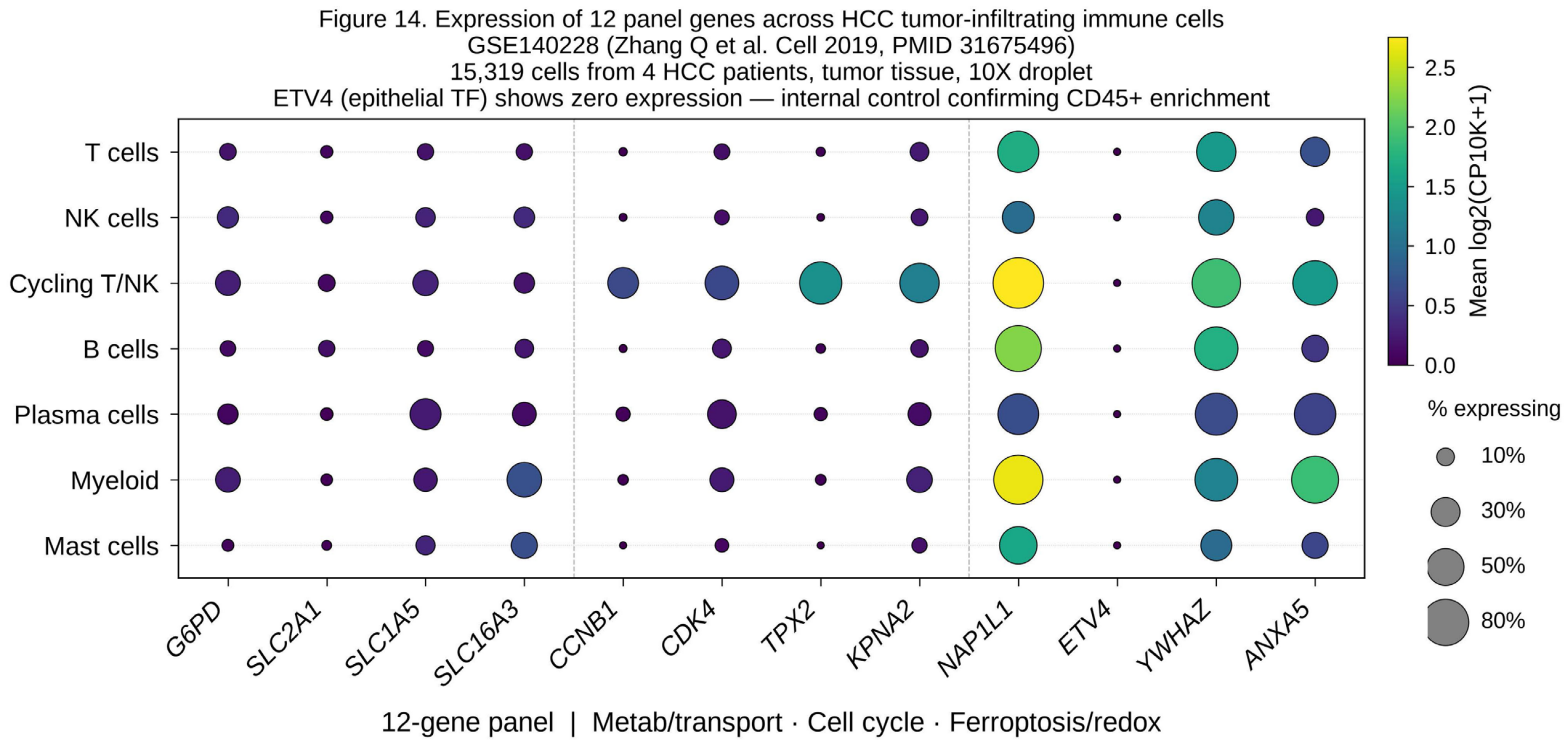
Single-cell validation of the 12 signature genes in the HCC immune microenvironment (GSE140228, retrieved through TISCH2). Dot plot showing the expression of the 12 risk-model genes (G6PD, SLC2A1, SLC1A5, SLC16A3, CCNB1, CDK4, TPX2, KPNA2, NAP1L1, ETV4, YWHAZ, ANXA5) across seven major immune-cell compartments (T cells, NK cells, cycling T/NK, B cells, plasma cells, myeloid cells, mast cells). Dot size encodes the percentage of cells expressing each gene; dot colour encodes the mean log2(CP10K + 1) expression. Genes are grouped by functional layer (metabolism/transport, cell cycle, ferroptosis/redox). ETV4 shows near-absent expression in immune cells, consistent with its tumour-cell-intrinsic role.

To resolve which immune-cell compartments express the signature genes, we examined the HCC tumour-infiltrating immune-cell atlas GSE140228^32^ (15,319 cells from four donors, retrieved through TISCH2^33^; Figure 14, Table S11). The cell-cycle genes *CCNB1*, *TPX2*, *KPNA2* and *CDK4* were predominantly expressed in cycling T/NK cells, the metabolic/redox genes *G6PD*, *SLC2A1* and *SLC1A5* in NK and myeloid cells, and the transcription factor *ETV4* was virtually absent from all immune compartments, consistent with its tumour-cell-intrinsic role. A more granular annotation across 28 finer subsets is provided in Figure S7 and Table S11.

## Discussion

Herein, we show that genes related to ferroptosis and metabolic reprogramming have clear prognostic value in HCC. These genes were differentially expressed between tumor and healthy liver tissues and also distinguished two molecular subtypes of HCC with distinct clinical features and survival outcomes. The subtypes differed not only in prognostic outcomes but also in immune activity and mutation-associated features. According to these findings, we developed a prognostic risk model using FPMRRGs, which demonstrated excellent predictive performance for 1/3/5-year overall survival. Together, these results suggest that this group of genes may function as biomarkers to stratify risk and as potential therapeutic targets.

The 12 genes included in our model are associated with numerous biological processes known to contribute to HCC progression. For example, SLC1A5, SLC2A1, and G6PD influence cellular metabolism and redox balance, thereby indirectly regulating susceptibility to ferroptosis through effects on amino acid and glucose metabolism, as well as NADPH and glutathione levels 34-37. ANXA5 has been linked to apoptosis, epigenetic regulation, and membrane transport, as well as tumor invasion, angiogenesis, and immune cell infiltration in HCC 38-42. NAP1L1 promotes tumor progression through multiple mechanisms, including ubiquitination of BIRC2, activation of Wnt signaling, and facilitation of cell cycle progression, as well as recruitment of transcriptional regulators such as HDGF/c-Jun 43-45. Other genes in the model further highlight key aspects of tumor biology. SLC16A3 has been associated with immune suppression and resistance to therapy 46, 47, while KPNA2 plays a vital role in telomere maintenance and tumor progression 48. CCNB1 contributes to cell cycle regulation and lipid metabolism through the FOXM1-CCNB1 axis 49, 50, and TPX2 has been linked to tumor proliferation, immune evasion, and resistance to sorafenib 51, 52. The CDK4/6-DUB3 axis stabilizes YAP1, a key effector of the Hippo pathway that promotes tumor growth 53. ETV4 is involved in hepatic inflammation and angiogenesis 54, 55, and YWHAZ participates in multiple signaling pathways that are dysregulated in HCC 56, 57. Compared with traditional classification systems based primarily on proliferative status or mutation profiles, this gene set captures additional dimensions of tumor biology, including metabolic reprogramming, ferroptosis sensitivity, and immune regulation. This provides a more integrated view of HCC heterogeneity and may offer new opportunities for both diagnosis and treatment.

Several gene expression-based models for HCC have been reported. For instance, a 10-gene LASSO-based model validated across TCGA and ICGC cohorts showed enrichment of cell cycle-related pathways in high-risk patients and metabolic pathways in low-risk patients 58. Tang et al. 59 developed a four-gene model focused on ferroptosis and iron metabolism, in which the high-risk group demonstrated greater tumor mutational burden, increased immune infiltration, and elevated immune checkpoint expression. Similarly, Wu et al. 60 proposed a five-gene ferroptosis-related model that showed strong predictive performance across multiple datasets, with high-risk patients displaying features of immune escape and higher TIDE scores. Most existing models, however, are derived from broad or relatively heterogeneous gene sets. In contrast, our model specifically integrates ferroptosis and metabolic reprogramming, two closely linked processes in tumor biology. Based on this framework, HCC can be broadly divided into subtypes characterized by features such as ferroptosis tolerance with high glycolytic activity and limited immune infiltration, versus a more ferroptosis-sensitive state with lower metabolic activity and greater immune engagement. From a biological perspective, this model emphasizes pathways related to nutrient transport, glycolysis and the pentose phosphate pathway, ferroptosis regulation, and epigenetic control.

Our findings suggest that interconnected metabolic, hypoxic, and lipid signaling pathways may collectively determine whether tumor cells adopt a ferroptosis-sensitive or ferroptosis-tolerant state. This aligns with prior reports showing that these pathways play central roles in HCC development and progression. In particular, we identified four key signaling axes: the HIF-1-driven glycolysis and central carbon metabolism pathway, the PPAR-regulated fatty acid and cholesterol metabolism pathway, the bile acid-cholesterol-inflammation network, and the ROS/redox-ferroptosis regulatory system. These pathways appear to coordinate ferroptosis resistance, metabolic reprogramming, and tumor growth. For example, activation of HIF-1 promotes glycolysis through upregulation of glucose transporters and key glycolytic enzymes such as HK2 61. The SOX4-ChREBP-SCD1 axis drives the synthesis of monounsaturated fatty acids, which can suppress ferroptosis 62, while the SLC7A11-GSH-GPX4 axis plays a central role in controlling cellular sensitivity to ferroptosis 63, 64. In addition, these pathways can reshape the tumor microenvironment by increasing the production of metabolites such as lactate and bile acids, as well as inflammatory mediators, thereby promoting immune modulation and angiogenesis 62, 65, 66. Importantly, alterations in these pathways are closely associated with clinical features of HCC. Enhanced activation of immune-metabolic pathways is often associated with an immunosuppressive microenvironment, characterized by enrichment of regulatory T cell (Treg) and myeloid-derived suppressor cell (MDSC), as well as upregulated expression of immune checkpoints (e.g., PD-1/PD-L1). These changes are also linked to resistance to commonly used therapies, including sorafenib and lenvatinib, through mechanisms involving metabolic rewiring and fatty acid metabolism 67-69. Taken together, these findings provide a rationale for exploring stratified therapeutic strategies in HCC patients defined by the 12-gene ferroptosis-metabolic reprogramming signature. High-risk tumors exhibited coordinated upregulation of glycolytic and HIF-1 signaling pathways together with an immunosuppressive immune microenvironment, characterized by elevated T-cell exhaustion, Treg, and M2 TAM signatures and reduced cytotoxicity (Figure 13 and Table S10). These features suggest that high-risk patients may be particularly vulnerable to therapeutic approaches that simultaneously target metabolic vulnerabilities and relieve immune suppression. Biologically plausible strategies for this subgroup include combining immune checkpoint inhibitors with agents that modulate glycolysis or redox balance (such as glycolysis or pentose-phosphate pathway inhibitors) or that enhance ferroptosis sensitivity through lipid metabolism regulation (such as SCD1 inhibitors). In contrast, low-risk patients, who displayed lower glycolytic activity and less pronounced immunosuppressive features, may derive sufficient benefit from standard immune checkpoint inhibitor-based regimens without the added complexity of multi-drug combinations. These hypotheses are grounded in the pathway enrichment and functional immune profiling results of the present study; however, they remain to be tested in prospective clinical trials and functional experiments.

Our immune-related analyses further highlight the clinical relevance of HCC subtype classification. Immune checkpoint activity and immune cell infiltration are closely linked and play a central role in shaping treatment response. ICGs (e.g., PD-1/PD-L1), are well-established therapeutic targets, and their inhibition can restore anti-tumor immunity 70, 71. Moreover, the immune infiltration profile of the tumor microenvironment is an important determinant of prognosis and response to immunotherapy 72, 73. Consistent with these concepts, we observed significant differences in ICG expression between the two HCC subtypes. Key checkpoint molecules, including LAG3, CTLA4, and PD-L1 (CD274), were more highly expressed in Subtype 1, which also showed higher TIDE scores and lower effector cell activity. This pattern suggests a more immunosuppressive phenotype, potentially associated with increased immune evasion and reduced responsiveness to immunotherapy. In contrast, Subtype 2 displayed a less immunosuppressive profile, indicating a greater likelihood of benefiting from immune checkpoint inhibitors (ICIs). These findings suggest that integrating our prognostic model with immune-related indicators, such as ICG expression and TIDE scores, could help guide individualized treatment strategies. In practice, this approach may support decisions on whether to use ICIs alone, combine ICIs with tyrosine kinase inhibitors, incorporate metabolic-targeted therapies, or avoid immunotherapy in patients unlikely to benefit.

Beyond cell-type abundance, the functional immune profile of the tumour microenvironment reinforces the immune-evasive phenotype of high-risk patients: their elevated exhaustion/Treg/MDSC signatures and reduced effector activity converge on a "cold" or immunosuppressive tumour state that may limit responses to single-agent immune checkpoint inhibitors, while the same patients could potentially benefit from combinations that target the suppressive compartment (e.g., MDSC- or Treg-depleting agents) alongside ICIs. The single-cell mapping further suggests that the cell-cycle genes in our model partly reflect tumour-infiltrating proliferating lymphocytes rather than tumour-cell proliferation alone, while the redox gene *G6PD* shows expression in NK cells linked to interferon response — an observation that warrants experimental follow-up in HCC immune biology.

This study has several limitations. Firstly, our findings are based entirely on *in silico* analyses and have not yet been validated in experimental or clinical settings. Although we included relatively large cohorts from the TCGA and GEO databases, residual batch effects and heterogeneity in data processing may limit the broader applicability of the results. In addition, we did not account for other factors, such as environmental exposures, lifestyle variables, or additional genetic alterations, that may influence HCC progression and could confound the observed associations. These limitations highlight the need for further validation in independent cohorts and in clinical tissue samples. Future studies should also incorporate functional experiments to better define the functional role of the identified genes. Additionally, the permissive |log2FC| > 0.25 threshold used for initial DEG selection may have included weakly-changing transcripts; a sensitivity analysis (Table S6, Figure S6) confirmed that the headline 12-gene signature is recoverable at this cutoff but that the candidate gene pool shrinks substantially at |log2FC| > 1. Direct comparison against BCLC staging in TCGA-LIHC was not possible because BCLC stage is not provided in the TCGA clinical files; the head-to-head comparison was therefore performed against AJCC TNM in TCGA and against BCLC in GSE76427. Finally, several important limitations of the immune and single-cell analyses should be noted. The functional immune signature scoring (Figure 13) and single-cell expression mapping (Figure 14) are entirely *in silico*. GSE140228 is a CD45+-enriched dataset that does not include malignant cells and comprises only four donors; therefore, the cellular localization findings require validation in larger, unsorted HCC cohorts with paired tumour and immune compartments. Additionally, the head-to-head comparison against BCLC staging in GSE76427 was limited by the small number of overall survival events (n = 23), resulting in wide confidence intervals for NRI and IDI estimates; these results should be interpreted with caution and ideally validated in larger external cohorts.

## Conclusion

In summary, we identified a set of key genes associated with ferroptosis and metabolic reprogramming in HCC and established a prognostic model with good predictive performance. This model provides new insight into the molecular basis of HCC and highlights potential biomarkers for patient stratification and personalized treatment. Further work integrating clinical validation with mechanistic studies will be important to confirm these findings and to examine the therapeutic potential of targeting these pathways.

## Supplementary Material

Supplementary figures and table legends.

Supplementary tables.

## Ethics Committee Approval and Patient Consent

This research was based entirely on publicly available datasets and did not involve the acquisition of new human/animal data. Ethical approval was therefore not required.

## Figures and Tables

**Table 1:** GEO dataset information.

	GSE10143	GSE76427
Platform	GPL5474	GPL10558
Species	*Homo sapiens*	*Homo sapiens*
Tissue	Liver cancer	Liver cancer tumor tissue
LIHC group	80	115
Control group	307	52
Reference	PMID:31344396	PMID:29117471

LIHC, liver cancer.

**Table 2:** Clinical features of liver cancer patients in the TCGA-LIHC dataset.

Characteristic	Subtype 1	Subtype 2	*P*-value
n	113	256	
OS, n (%)			< 0.001
1	59 (16%)	71 (19.2%)	
0	54 (14.6%)	185 (50.1%)	
OS.time, median (IQR)	382 (171, 765)	666.5 (404.5, 1354)	< 0.001
Age, median (IQR)	61 (51, 68)	62 (52, 69)	0.554
Gender, n (%)			0.205
Male	71 (19.2%)	178 (48.2%)	
Female	42 (11.4%)	78 (21.1%)	
DSS, n (%)			0.014
1	33 (9.1%)	46 (12.7%)	
0	77 (21.3%)	205 (56.8%)	
DSS.time, median (IQR)	382 (171, 765)	666.5 (404.5, 1354)	< 0.001
PFI, n (%)			0.504
1	59 (16%)	124 (33.6%)	
0	54 (14.6%)	132 (35.8%)	
PFI.time, median (IQR)	233 (102, 408)	463.5 (200.5,776)	< 0.001
Stage_Pathologic, n (%)			< 0.001
I	32 (9.3%)	140 (40.6%)	
II	32 (9.3%)	53 (15.4%)	
III	40 (11.6%)	43 (12.5%)	
IV	1 (0.3%)	4 (1.2%)	
Stage_M, n (%)			1.000
M0	84 (31.2%)	181 (67.3%)	
M1	1 (0.4%)	3 (1.1%)	
Stage_N, n (%)			0.790
N0	78 (30.6%)	173 (67.8%)	
N1	2 (0.8%)	2 (0.8%)	
Stage_T, n (%)			< 0.001
T1	33 (9%)	149 (40.7%)	
T2	39 (10.7%)	54 (14.8%)	
T3	36 (9.8%)	42 (11.5%)	
T4	5 (1.4%)	8 (2.2%)	

OS, overall survival; IQR, interquartile range; DSS, Disease-Specific Survival; PFI, Progression-Free Interval.

**Table 3:** GSEA enrichment analysis.

ID	Enrichment Score	NES	*P*-value	P.adjust	*q*-value
CHIANG_LIVER_CANCER_SUBCLASS_PROLIFERATION_DN	0.958110140055881	4.01382390973097	1e-10	2.86291079812207e-09	1.73610081541883e-09
DESERT_PERIPORTAL_HEPATOCELLULAR_CARCINOMA_SUBCLASS_UP	0.92084289780541	3.7436911901007	1e-10	2.86291079812207e-09	1.73610081541883e-09
LEE_LIVER_CANCER_SURVIVAL_UP	0.893677727435758	3.72581224464666	1e-10	2.86291079812207e-09	1.73610081541883e-09
WOO_LIVER_CANCER_RECURRENCE_DN	0.935544397759437	3.64602563131129	1e-10	2.86291079812207e-09	1.73610081541883e-09

**Table 4:** Functional and pathway enrichment analyses of FPMRRDEGs.

Ontology	ID	Description	GeneRatio	BgRatio	*P*-value	*P*.adjust
BP	GO:0070542	Response to fatty acid	6/52	63/18800	2e-08	3.89e-05
BP	GO:0001666	Response to hypoxia	9/52	286/18800	7.99e-08	7.46e-05
BP	GO:0036293	Response to decreased oxygen levels	9/52	299/18800	1.17e-07	7.46e-05
BP	GO:0009410	Response to xenobiotic stimulus	10/52	411/18800	1.56e-07	7.46e-05
BP	GO:0072593	Reactive oxygen species metabolic process	8/52	231/18800	2.17e-07	7.46e-05
CC	GO:0072562	Blood microparticle	7/52	147/19594	1.17e-07	2.13e-05
CC	GO:0031965	Nuclear membrane	7/52	300/19594	1.36e-05	0.0012
CC	GO:0005635	Nuclear envelope	8/52	479/19594	3.52e-05	0.0021
CC	GO:0009925	Basal plasma membrane	6/52	251/19594	5.17e-05	0.0024
CC	GO:0045178	Basal part of cell	6/52	269/19594	7.59e-05	0.0028
MF	GO:0005504	Fatty acid binding	4/52	49/18410	1.09e-05	0.0018
MF	GO:0005324	Long-chain fatty acid transporter activity	3/52	17/18410	1.41e-05	0.0018
MF	GO:0033293	Monocarboxylic acid binding	4/52	81/18410	8.02e-05	0.0067
MF	GO:0016829	Lyase activity	5/52	195/18410	0.0002	0.0138
MF	GO:0016538	Cyclin-dependent protein serine/threonine kinase regulator activity	3/52	50/18410	0.0004	0.0178
KEGG	hsa04066	HIF-1 signaling pathway	6/45	109/8164	2.63e-05	0.0020
KEGG	hsa00010	Glycolysis/gluconeogenesis	5/45	67/8164	3.03e-05	0.0020
KEGG	hsa05230	Central carbon metabolism in cancer	5/45	70/8164	3.75e-05	0.0020
KEGG	hsa03320	PPAR signaling pathway	5/45	75/8164	5.25e-05	0.0021
KEGG	hsa01200	Carbon metabolism	5/45	115/8164	0.0004	0.0127

**Table 5:** Results of GSVA.

ID	logFC	AveExpr	t	P-value	P.adjust	B
HALLMARK_BILE_ACID_METABOLISM	0.415583108846574	0.026486158771888	12.6799821272068	4.85129345429068e-31	1.21282336357267e-29	59.5951046550037
HALLMARK_XENOBIOTIC_METABOLISM	0.353528389269362	0.0184061819616987	10.9892662321922	1.26138977950517e-24	1.26138977950517e-23	44.9411076248228
HALLMARK_FATTY_ACID_METABOLISM	0.327565698011563	0.010576269173411	10.5569573401611	4.66497591885173e-23	3.33212565632266e-22	41.3636683463416
HALLMARK_COAGULATION	0.298034361804666	-0.000887601	9.59323404078445	1.09265936397131e-19	5.46329681985655e-19	33.6841193800371
HALLMARK_PEROXISOME	0.257558062034217	0.0108770824934781	8.96711056754557	1.3321613121176e-17	6.05527869144363e-17	28.9372554740285
HALLMARK_ADIPOGENESIS	0.244026632483944	0.00171678149728811	8.65583845460858	1.34453686794731e-16	5.60223694978046e-16	26.6551922198714
HALLMARK_PANCREAS_BETA_CELLS	0.24378243903967	0.0669493888696586	10.0290272102809	3.44630800144363e-21	1.91461555635757e-20	37.1036773726728
HALLMARK_KRAS_SIGNALING_DN	0.20586315057562	0.0393441176016757	10.9053726545663	2.55670196851572e-24	2.1305849737631e-23	44.2408982069883
HALLMARK_OXIDATIVE_PHOSPHORYLATION	0.1886500786541	-0.0308489	5.14367945740452	4.29512666289033e-07	1.19309073969176e-06	5.22106714635928
HALLMARK_HEME_METABOLISM	0.113413190464048	-0.013221307	4.94011963936482	1.1653004347879e-06	3.0665800915471e-06	4.25836259601629
HALLMARK_DNA_REPAIR	-0.163704654	-0.031274239	-5.604628483	3.97834532111603e-08	1.17010156503413e-07	7.52505623068901
HALLMARK_MYC_TARGETS_V2	-0.174267198	-0.038933623	-4.563565463	6.76892513127351e-06	1.69223128281838e-05	2.56835203723879
HALLMARK_PROTEIN_SECRETION	-0.178083702	-0.024433637	-5.956425316	5.80784734120289e-09	1.8149522941259e-08	9.3960183902469
HALLMARK_UNFOLDED_PROTEIN_RESPONSE	-0.222851446	-0.046817803	-8.574969179	2.4303007545938e-16	9.34731059459153e-16	26.0711588968652
HALLMARK_MTORC1_SIGNALING	-0.223011603	-0.025790816	-8.510872607	3.87527946813091e-16	1.38402838147532e-15	25.6109032299221
HALLMARK_PI3K_AKT_MTOR_SIGNALING	-0.25484997	-0.04076783	-11.86745846	6.61677578493636e-28	8.27096973117045e-27	52.431092044599
HALLMARK_MYC_TARGETS_V1	-0.269522872	-0.027308494	-7.842362427	4.37092071234575e-14	1.45697357078192e-13	20.9549650479978
HALLMARK_MITOTIC_SPINDLE	-0.299221046	-0.025557915	-10.35012387	2.55371246855685e-22	1.59607029284803e-21	39.6799427534636
HALLMARK_G2M_CHECKPOINT	-0.4281277	-0.028450989	-13.25915687	2.51662096110547e-33	1.25831048055274e-31	64.8200596528356
HALLMARK_E2F_TARGETS	-0.43202263	-0.021096534	-12.1490037	5.55273953732687e-29	9.25456589554479e-28	54.8898355903733

GSVA, Gene Set Variation Analysis; AveExpr, average expression; logFC, log fold change; B, Bayes statistic.

**Table 6:** Clinical performance comparison of the 12-gene RiskScore with staging systems

Cohort	Stage_var	Endpoint	Model	N	Events	C_index	AUC_1y_3y_5y	NRI_5y	IDI_5y	Cmp_vs_StageOnly_p
TCGA-LIHC	AJCC TNM (I/II/III/IV)	OS	[TCGA] TNM stage only	339	116	0.660 [0.586-0.734]	0.660 / 0.673 / 0.628	—	—	—
TCGA-LIHC	AJCC TNM (I/II/III/IV)	OS	[TCGA] RiskScore only	339	116	0.710 [0.662-0.757]	0.818 / 0.713 / 0.690	—	—	0.103
TCGA-LIHC	AJCC TNM (I/II/III/IV)	OS	[TCGA] RiskScore + TNM stage	339	116	0.717 [0.668-0.765]	0.818 / 0.749 / 0.721	0.247 [0.035-0.442]	0.099 [0.034-0.170]	0.0386
GSE76427	BCLC (0/A vs B vs C)	OS	[TCGA-β] BCLC only	115	23	0.719 [0.556-0.881]	0.588 / 0.696 / 0.766	—	—	—
GSE76427	BCLC (0/A vs B vs C)	OS	[TCGA-β] RiskScore only	115	23	0.571 [0.435-0.706]	0.567 / 0.499 / 0.375	—	—	—
GSE76427	BCLC (0/A vs B vs C)	OS	[TCGA-β] RiskScore + BCLC	115	23	0.671 [0.549-0.793]	0.632 / 0.703 / 0.673	0.143 [-0.663-0.878]	-0.017 [-0.129-0.110]	0.839
GSE76427	BCLC (0/A vs B vs C)	OS	[Local] BCLC only	115	23	0.719 [0.556-0.881]	0.588 / 0.696 / 0.766	—	—	—
GSE76427	BCLC (0/A vs B vs C)	OS	[Local] RiskScore only	115	23	0.813 [0.740-0.886]	0.834 / 0.781 / 0.953	—	—	—
GSE76427	BCLC (0/A vs B vs C)	OS	[Local] RiskScore + BCLC	115	23	0.812 [0.740-0.883]	0.821 / 0.811 / 0.963	0.621 [0.004-1.003]	0.374 [0.050-0.632]	0.113

Footnotes: 1 TCGA-LIHC: AJCC pathologic stage simplified to I/II/III/IV (Stage IIIA/IIIB/IIIC merged into III; Stage IV/IVA/IVB into IV). Stage IV n=4 - wide HR CI expected. 2 TCGA-LIHC analytic n=339 of 363 with complete AJCC stage (24 NA-stage tumors excluded). 3 GSE76427: BCLC stage 0 (n=4) merged with A as both are surgery-eligible per BCLC clinical guidelines; final grouping 0/A (n=78) B (n=28) C (n=9). 4 GSE76427 OS events=23 (20% event rate); NRI/IDI 95% CIs are wide and should be interpreted with caution at this sample size. 5 Two RiskScore versions for GSE76427: [TCGA-beta] uses TCGA-trained Cox beta transferred to z-scored GSE76427 expression; [Local] refits Cox on GSE76427 z-scored expression. The [TCGA-beta] OS HR direction inverts (HR=0.72 NS) - known cross-platform coefficient-transfer failure. [Local] (HR=13.12 [3.70-46.53] p<0.001) shows the 12-gene panel carries independent prognostic information. 6 C-index: survcomp::concordance.index (Noether SE). Paired C-comp: survcomp::cindex.comp. NRI/IDI at 5y: survIDINRI::IDI.INF (npert=500). Time-AUC: timeROC. 7 AUC time horizons: TCGA-LIHC 365/1095/1825 days; GSE76427 1/3/5 years (native). Abbreviations: TNM=AJCC tumor-node-metastasis; BCLC=Barcelona Clinic Liver Cancer; RS=RiskScore; NRI=continuous net reclassification improvement; IDI=integrated discrimination improvement; AUC=area under ROC.

## Data Availability

All data used in this research were derived from publicly available databases. Details of the bioinformatics analysis are available from the corresponding author upon reasonable request.

## Abbreviations

AUC: Area under the curve
BH: Benjamini-Hochberg
CDF: Cumulative distribution function
CI: Confidence interval
CDK4: Cyclin-dependent kinase 4
CTLA4: Cytotoxic T-lymphocyte associated protein 4
DCA: Decision curve analysis
DEGs: Differentially expressed genes
DSS: Disease-specific survival
CD274: Cluster of differentiation 274
FDR: False discovery rate
FOXM1: Forkhead box M1
FPMRRDEGs: Ferroptosis- and metabolic reprogramming-related differentially expressed genes
FPMRRGs: Ferroptosis- and metabolic reprogramming-related genes
GEO: Gene Expression Omnibus
GO: Gene ontology
GPX4: Glutathione peroxidase 4
GSH: Glutathione
GSVA: Gene set variation analysis
HCC: Hepatocellular carcinoma
HIF-1: Hypoxia-inducible factor-1
Hippo: Hippo signaling pathway
HR: Hazard ratio
ICIs: Immune checkpoint inhibitors
ICGs: Immune checkpoint genes
IDI: Integrated discrimination improvement
IPS: Immunophenoscore
IQR: Interquartile range
KEGG: Kyoto Encyclopedia of Genes and Genomes
KM: Kaplan-Meier
LAG3: Lymphocyte-activation gene 3
LASSO: Least absolute shrinkage and selection operator
LIHC: Liver hepatocellular carcinoma
MDSC: Myeloid-derived suppressor cell
MSI: Microsatellite instability
MSigDB: Molecular Signatures Database
NADPH: Nicotinamide adenine dinucleotide phosphate
NES: Normalized enrichment score
NRI: Net reclassification improvement
OS: Overall survival
PD-1: Programmed cell death protein 1
PD-L1: Programmed cell death ligand 1
PFI: Progression-free interval
PPAR: Peroxisome proliferator-activated receptor
ROC: Receiver operating characteristic
ROS: Reactive oxygen species
SD: Standard deviation
SLC: Solute carrier
ssGSEA: Single-sample gene set enrichment analysis
TCGA: The Cancer Genome Atlas
TIDE: Tumor immune dysfunction and exclusion
TMB: Tumor mutational burden
Treg: Regulatory T cell
t-SNE: t-distributed stochastic neighbor embedding
YAP1: Yes-associated protein 1
