# Supplementary material for: A Prognostic Risk Model for Hepatocellular Carcinoma Integrating Ferroptosis and Metabolic Reprogramming Signatures
Source: J Cancer. 2026 Jul 10;17(7):1295–317. doi: 10.7150/jca.135837 (PMC13410346; doi:10.7150/jca.135837)
Supplement: Supplementary file 1 — Supplementary figures and table legends. [file jcav17p1295s1.pdf]

Supplementary Figures

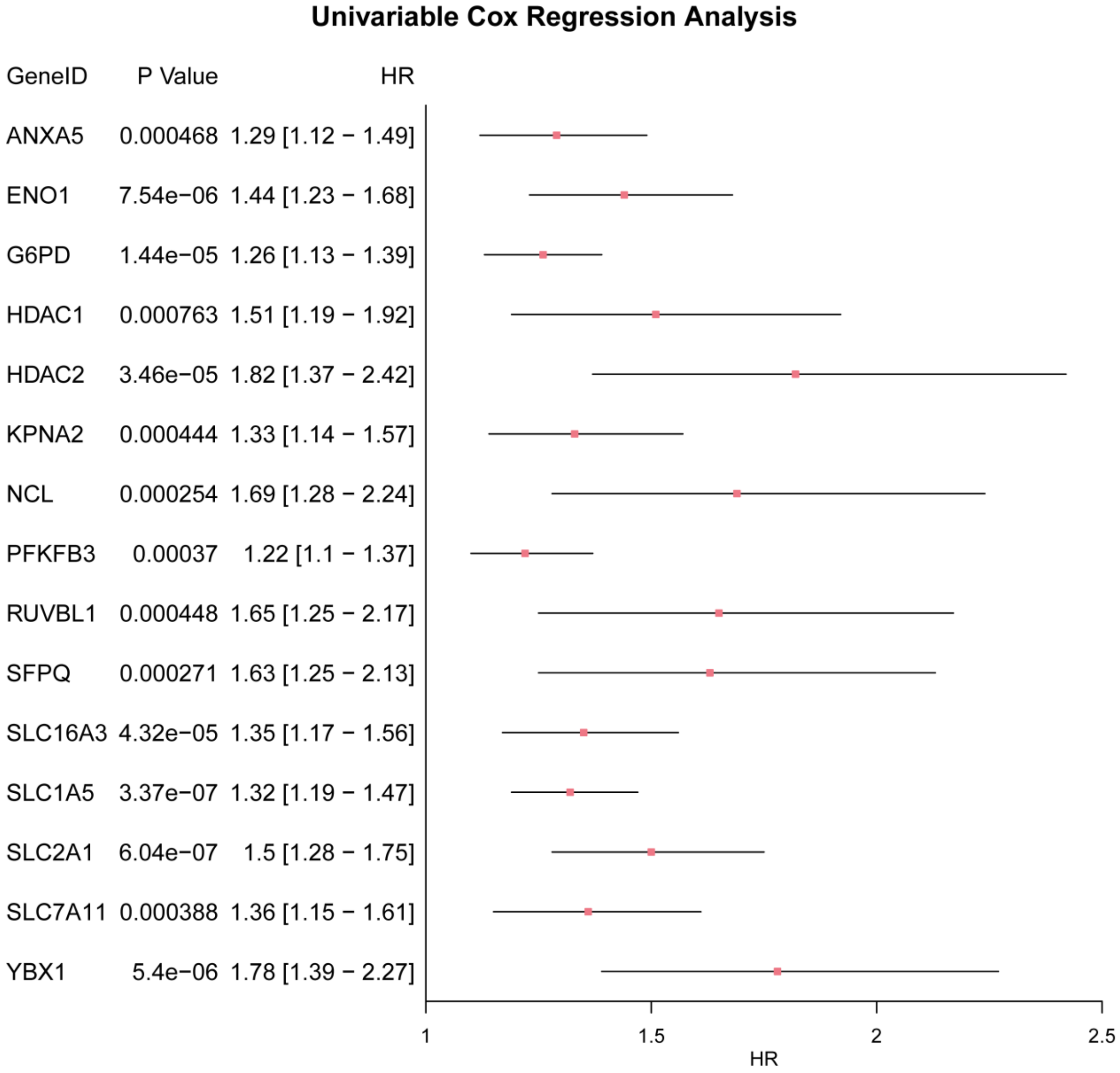

Supplementary Figure 1. Univariable analysis of prognosis-associated genes.

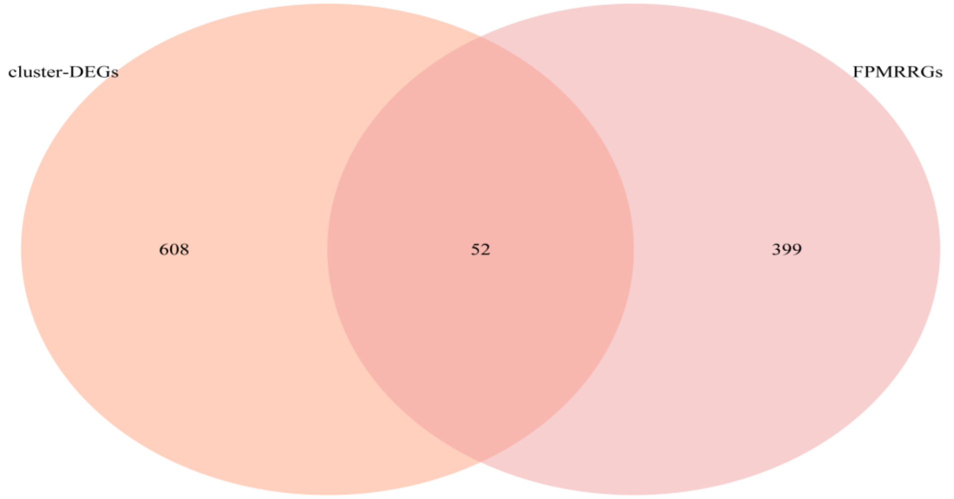

Supplementary Figure 2. Overlap between cluster-DEGs and FPMRRGs.

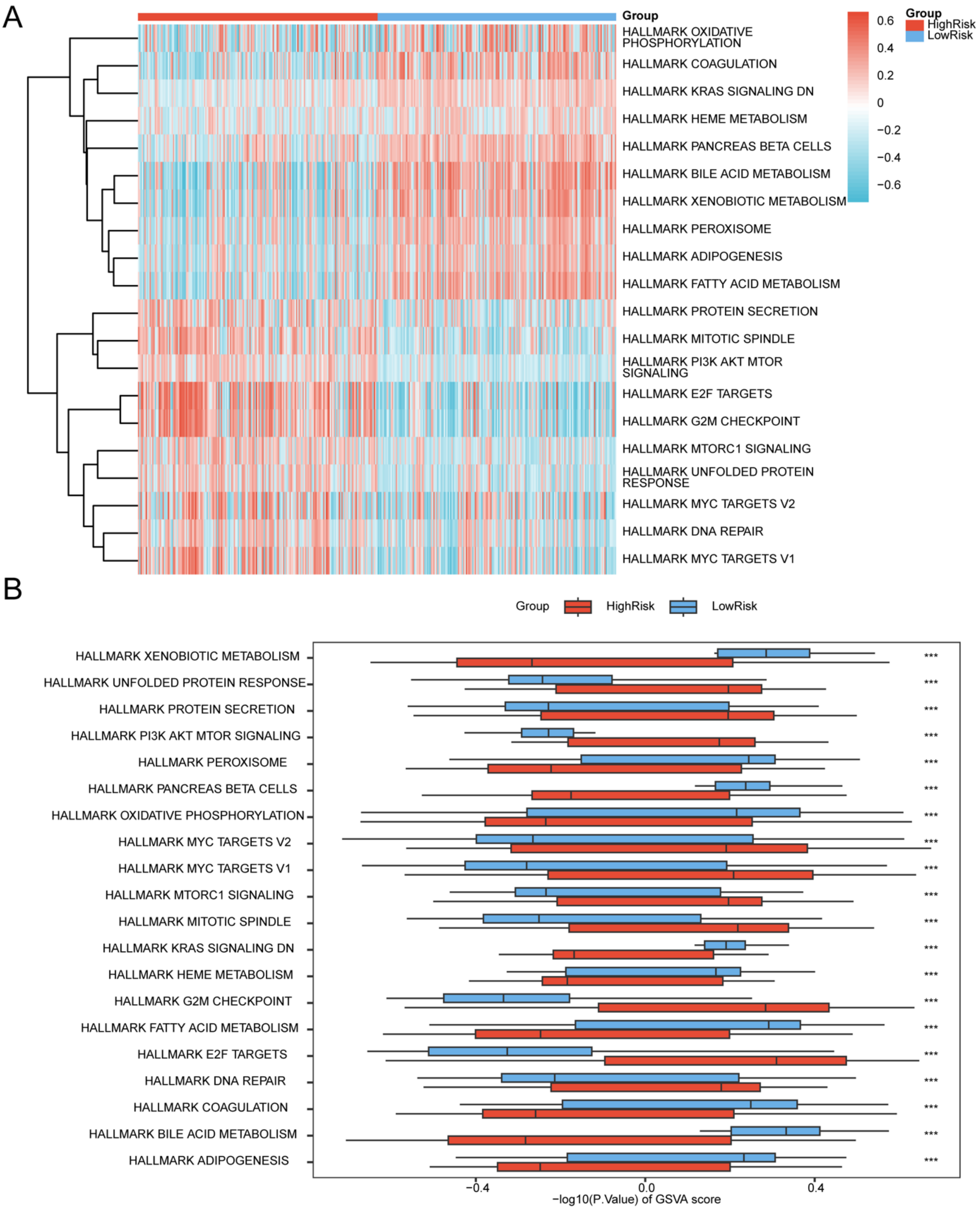

Supplementary Figure 3. GSVA analysis of low- and high-risk HCC groups. (A) Heatmap displaying pathway enrichment differences between low- and high-risk groups in TCGA-LIHC. (B) Group comparison plot illustrating significantly enriched pathways. Significance levels: ns ($p \geq 0.05$), *$p < 0.05$, **$p < 0.01$, and ***$p < 0.001$.

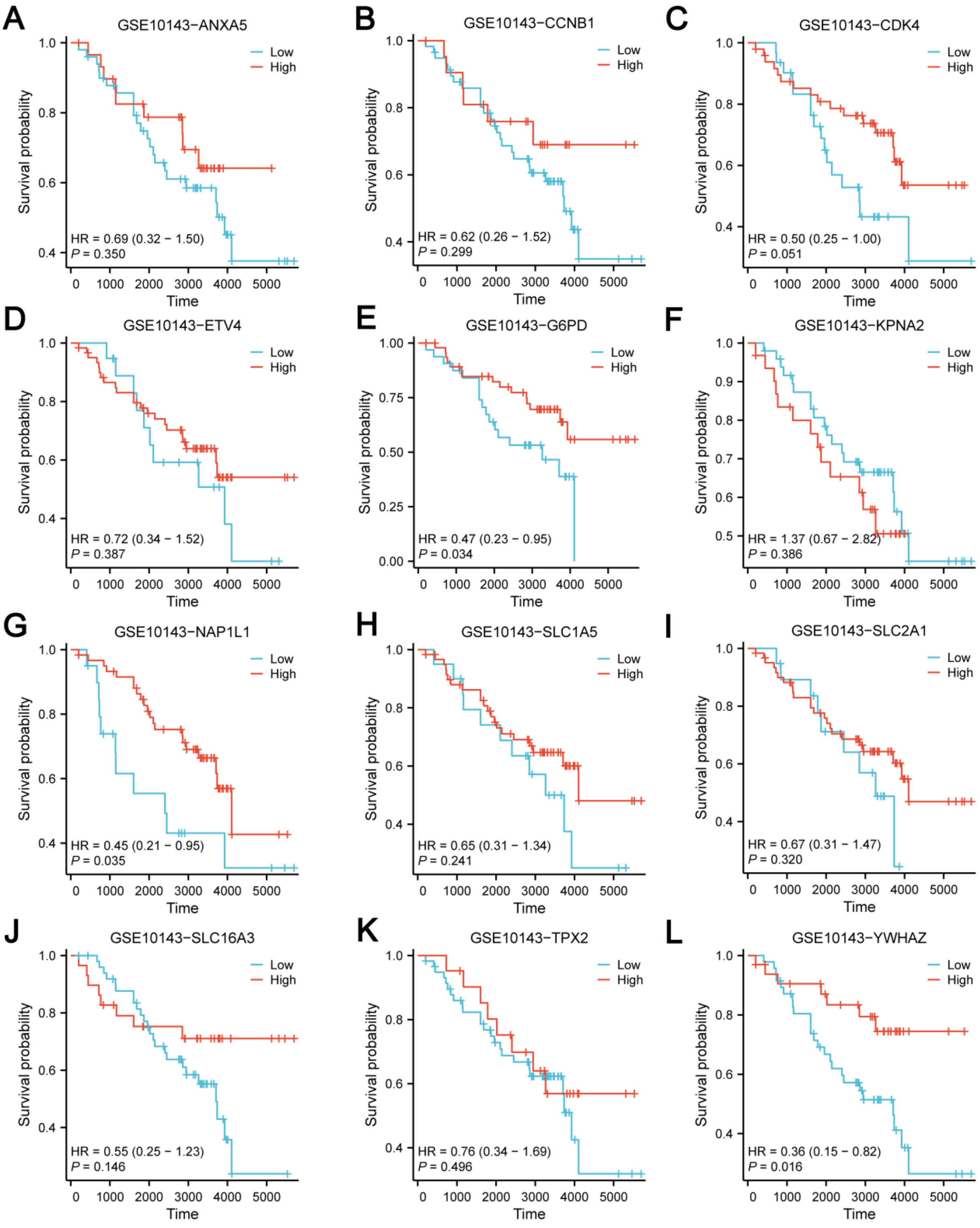

Supplementary Figure 4. KM analysis for GSE10143. KM curves showing the associations among key gene expression levels and overall survival in HCC patients.

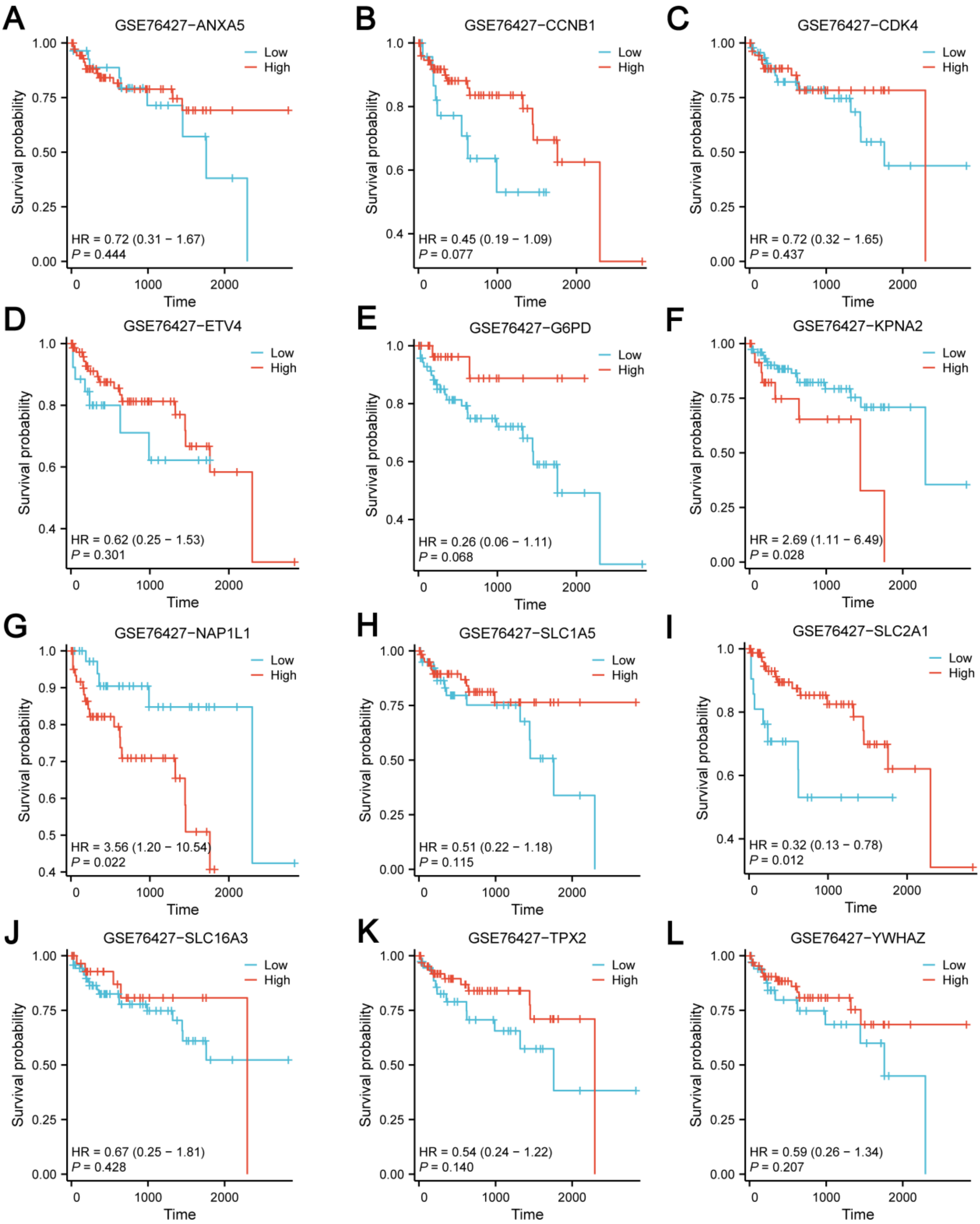

Supplementary Figure 5. KM analysis for GSE76427. KM curves illustrating the relationships among key gene expression and overall survival in HCC patients.

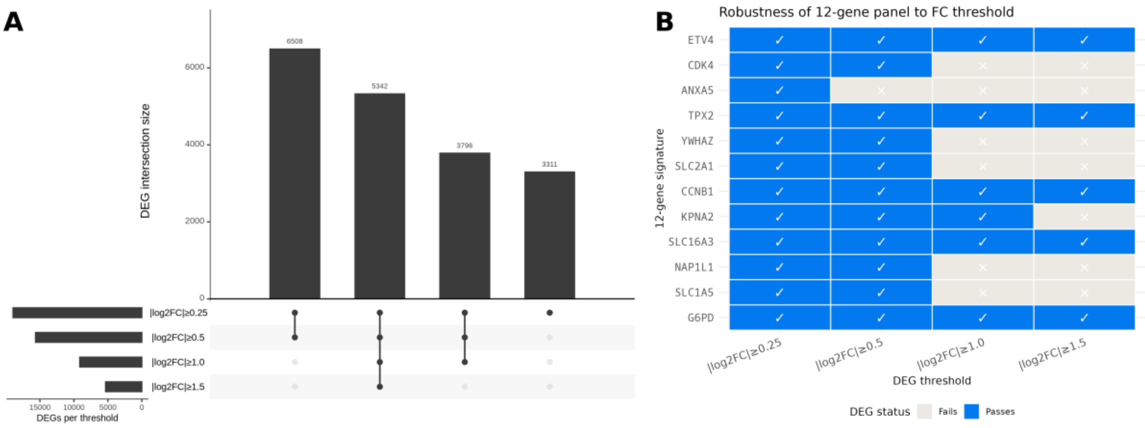

Supplementary Figure 6. Robustness of the FPMRRDEG pool across fold-change thresholds. (A) UpSet plot showing intersections of FPMRRDEGs at four $|log_2FC|$ cutoffs (>0.25, >0.5, >1.0, >1.5), with 12/12, 11/12, 6/12, and 5/12 signature genes retained at each threshold. (B) Heatmap showing retention status of the 12 signature genes across cutoffs. ANXA5 is excluded at $|log_2FC| > 0.5$; 6 genes pass $|log_2FC| > 1.0$, and 5 genes pass $|log_2FC| > 1.5$. C-indices of the Cox model with threshold-matched gene subsets are 0.710, 0.712, 0.698, and 0.694, respectively.

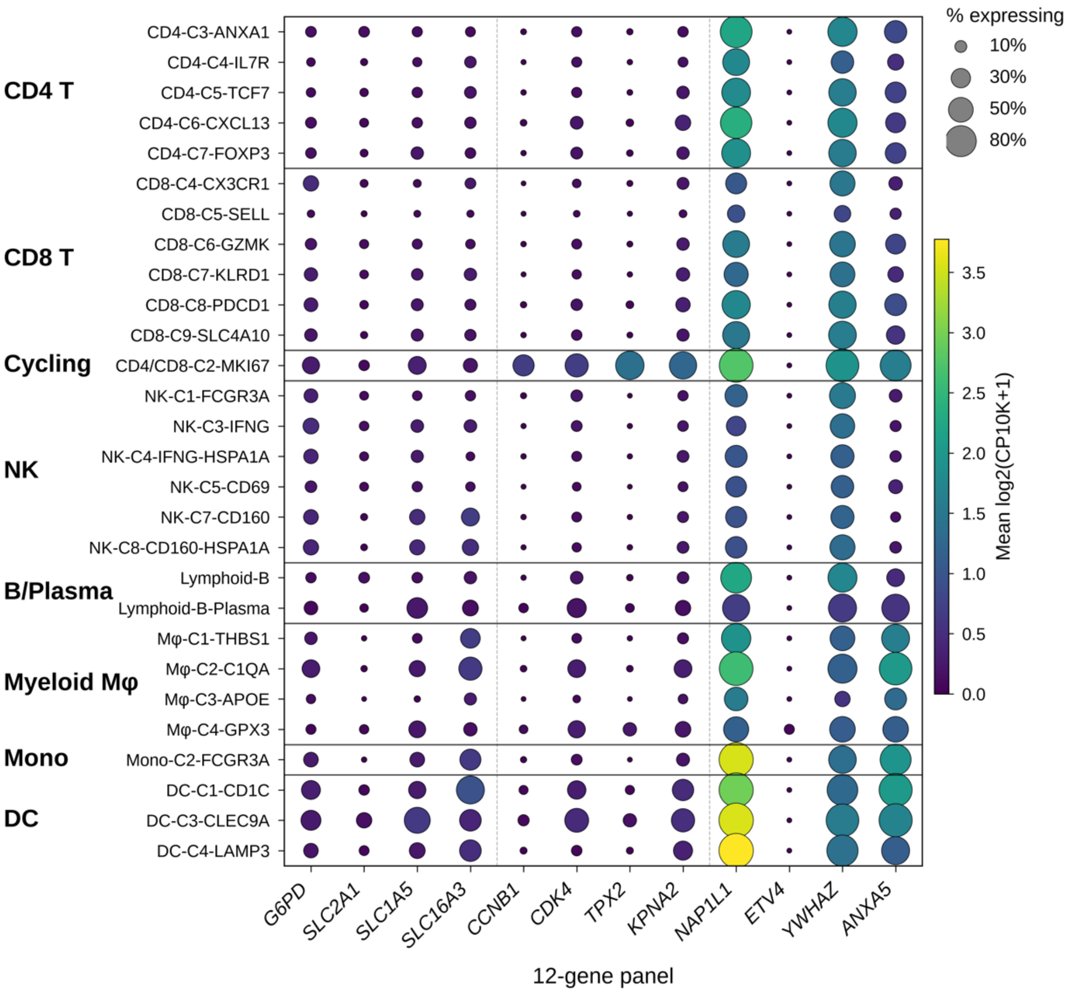

Supplementary Figure 7. Single-cell expression of the 12-gene signature in HCC immune subtypes. Dot plot showing expression of the 12 signature genes across 28 fine immune subtypes in GSE140228 (15,319 tumour cells from 4 donors). Dot size indicates the percentage of expressing cells; colour indicates mean $log_2(CP10K+1)$ expression. Subtypes

are grouped by major lineages (T, NK, B, plasma, myeloid, mast cells). ETV4 is absent in all immune subtypes, confirming epithelial-restricted expression.

Supplementary Tables

Table S1. Merged lists of ferroptosis-related genes (FPRGs) and metabolic reprogramming-related genes (MRRGs).
Complete lists of FPRGs (n=2,023) and MRRGs (n=1,951) compiled from GeneCards and literature. Includes Gene Symbol, Description, Uniprot ID, Relevance score, and GeneCards link. A column "Source_List" distinguishes the two source lists (777 genes overlap between them). These lists were intersected with TCGA-LIHC DEGs to obtain the 451 FPMRRGs used in subsequent analyses.

Table S2. List of 47 immune checkpoint genes (ICGs).
Curated list of 47 ICGs used to compare expression differences between the two HCC molecular subtypes.

Table S3. FPMRRDEGs significantly associated with survival (univariate Cox, $p<0.001$).
Fifteen FPMRRDEGs with $p<0.001$ from univariate Cox regression in TCGA-LIHC, including hazard ratios (1.22–1.82) and 95% confidence intervals. These genes were used for consensus clustering to define molecular subtypes.

Table S4. Differentially expressed genes between the two HCC subtypes.
DEGs identified by DESeq2 ($|\log2FC| > 1$, $p < 0.05$) between Subtype 1 and Subtype 2 in TCGA-LIHC. Used for GSEA and functional enrichment analyses.

Table S6. Sensitivity of the 12-gene signature to different |log2FC| thresholds.
Retention status of the 12 signature genes at |log2FC| cutoffs of 0.25, 0.5, 1.0, and 1.5. All 12 genes are retained at the published threshold ($|\log2FC| > 0.25$).

Table S6b. Apparent C-index of refitted Cox models at different |log2FC| thresholds.
Performance of Cox models using genes retained at each threshold: 12 genes (C-index 0.7098), 11 genes (0.7116), 6 genes (0.6977), and 5 genes (0.6937).

Table S7. Internal validation of the 12-gene RiskScore.
Apparent C-index (0.7098), 10-fold cross-validation results (mean C-index 0.6760), and bootstrap-corrected C-index (0.6788, optimism 0.0309) in TCGA-LIHC.

Table S7b. Proportional hazards assumption test (Schoenfeld residuals).
Individual and global test results for the multivariate Cox model. Global test $p = 0.171$ (overall model valid despite some individual covariate violations).

Table S8. Multi-cohort validation of the 12-gene RiskScore.
Performance in TCGA-LIHC (training) and external GEO cohorts (GSE10143, GSE76427) using TCGA-derived vs locally refitted coefficients (HR, C-index, and time-dependent AUCs).

Table S9. Net benefit of the 12-gene RiskScore in decision curve analysis.
Net benefit values and gains of RiskScore (alone or combined with staging) over AJCC TNM (TCGA) or BCLC (GSE76427) at various risk thresholds and time horizons.

Table S10. Functional immune signature scores in low- vs high-risk groups.
singscore results for 11 immune gene sets in TCGA-LIHC. High-risk tumors showed elevated suppressive signatures (Treg, T-exhaustion, M2 TAM) and reduced cytotoxicity (Wilcoxon test with BH adjustment).

Table S11. Single-cell expression of the 12-gene signature in GSE140228 immune cells.
Mean expression and percentage of expressing cells for the 12 genes across major immune compartments (T, NK, cycling T/NK, B, plasma, myeloid, mast) in HCC tumor-infiltrating CD45+ cells.

Note: Table S5 has been removed/merged during revision.
